# Individual Pause-and-Go Motion Is Instrumental to the Formation and Maintenance of Swarms of Marching Locust Nymphs

**DOI:** 10.1371/journal.pone.0101636

**Published:** 2014-07-02

**Authors:** Gil Ariel, Yotam Ophir, Sagi Levi, Eshel Ben-Jacob, Amir Ayali

**Affiliations:** 1 Department of Mathematics, Bar Ilan University, Ramat-Gan, Israel; 2 Department of Zoology, Faculty of Life Sciences, Tel Aviv University, Tel Aviv, Israel; 3 Sackler School of Physics and Astronomy, Tel Aviv University, Tel Aviv, Israel; 4 Center for Theoretical Biological Physics, Rice University, Houston, Texas, United States of America; 5 Sagol School of Neuroscience, Tel Aviv University, Tel Aviv, Israel; University of Maribor, Slovenia

## Abstract

The principal interactions leading to the emergence of order in swarms of marching locust nymphs was studied both experimentally, using small groups of marching locusts in the lab, and using computer simulations. We utilized a custom tracking algorithm to reveal fundamental animal-animal interactions leading to collective motion. Uncovering this behavior introduced a new agent-based modeling approach in which pause-and-go motion is pivotal. The behavioral and modeling findings are largely based on motion-related visual sensory inputs obtained by the individual locust. Results suggest a generic principle, in which intermittent animal motion can be considered as a sequence of individual decisions as animals repeatedly reassess their situation and decide whether or not to swarm. This interpretation implies, among other things, some generic characteristics regarding the build-up and emergence of collective order in swarms: in particular, that order and disorder are generic meta-stable states of the system, suggesting that the emergence of order is kinetic and does not necessarily require external environmental changes. This work calls for further experimental as well as theoretical investigation of the neural mechanisms underlying locust coordinative behavior.

## Introduction

From ancient times and still today, the fascinating phenomenon of locust swarms continues to threaten agriculture and challenge science. One of the main difficulties in predicting and controlling locust outbreaks is that of our insufficient understanding of the fundamental principles underlying locust swarming. The challenge lies in connecting or deciphering the dynamic interactions between the behavior of individual animals, the coordinated activity of crowds consisting of millions of animals, and the environment. From a theoretical point of view, locust swarming is a quintessential example of collective motion [1], [2], [3], bearing resemblance to the formation and dynamics of schools of fish [4], flocks of birds [5], [6], human crowds [7], cells and bacteria [8], and even artificial agents (swarming robots; [9]). Indeed, the emergence of novel group-level behaviors has been described in terms such as “swarm intelligence” [10], [11], or the “mind of the swarm” [12], referring to the congruence in behavior of swarms composed of very different individuals.

Locusts, nonetheless, retain a special fascination for both scientists and laymen. These short-horned grasshoppers demonstrate density-dependent polyphenism [13]. At high population density, locusts actively aggregate, forming large hopper bands or adult swarms. In particular, the vast groups of gregarious nymphs that march in unison offer an exceptional model for the study of animal collective behavior.

Recent laboratory and field experiments in the desert locust, *Schistocerca gregaria*, and the Australian plague locust, *Chortoicetes terminifera*, have suggested that collective movement is, as previously reported [14], highly dependent on the density of animals in the group, and is mediated by combinations of social pair-wise interactions such as avoidance, alignment, and attraction [15], [16]. It was also demonstrated that groups can switch direction without external perturbation. A dominant feature characterizing locust behavior is an intermittent, pause-and-go, walking pattern. This behavior was recently thoroughly studied in single isolated locusts by Bazazi et al. [17], who found that pause duration is correlated with a high probability of turning. This result suggests that pauses relate to instances in which individuals make a decision - in this case on direction. The pause-and-go walking pattern was also observed in our own field observations during a recent desert locust outbreak in Israel’s Negev desert in the spring of 2013 (Ayali et al., unpublished).

A putative mechanism for locust swarming and marching behavior was recently, suggested, based on an escape and pursuit strategy, and driven by cannibalism [17], [18], [19], [20]. The general point of view underlying this somewhat attractive model is that animals chase their peers (conspecifics) in order to eat them, while at the same time, they flee from others to avoid being eaten (but see [16]). Several additional models have been suggested to describe the emergence of collective behavior in marching locusts, such as Viscek-type models [21], [22], [23], in which individuals align themselves with their neighbors with some error (noise) and continuous intego-differential models [24], [25]. In the Viscek model and its variations [1], [3] the system can be in one of two phases-disordered or ordered - in which synchronization is either low or high, respectively. The transition between the phases depends on some parameters such as the amount of noise [1]. All previous models successfully predict that while in the ordered phase the swarm can be in one of several metastable states, which can be characterized by an order parameter (see below). However, the switch between order and disorder has been related to some change in external conditions, such as density [21] or the animals’ diet [19]. Moreover, these modeling attempts are largely heuristic and are somewhat unsuccessful in linking the physiological and sensory conditions of the animal and the social interactions they infer.

The current work was aimed at providing novel insights into the mechanisms responsible for locust coordinated behavior. The principal interactions leading to the emergence of order in swarms of marching locust nymphs was studied both experimentally, using small groups of marching locusts in the lab, and using computer simulations. Specifically, motivated by the experiments of Buhl et al. [15] and Bazazi et al. [17], here we focused on the instrumental role of individual pause-and-go motion in the formation and maintenance of the collective motion in swarms of marching locust nymphs. The major sensory triggers and fundamental mechanisms leading to collective behavior within the swarm were inferred from behavior analysis. Preliminary electrophysiological investigation of the processing of visual inputs, relevant to the dynamic interactions between animals in a marching swarm lend further support to our hypotheses. Assisted by simple models, we show that intermittent motion has a pivotal role in the development and evolution of order and disorder within the swarm, as every time an animal starts moving it makes a small decision on whether or not to join the crowd.

## Materials and Methods

### Animals

A colony of the desert locusts, *Schistocerca gregaria* (Forskål) were raised at the Department of Zoology, Tel Aviv University, Israel in a controlled temperature of 30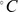 and 35–60% humidity, under 12D:12L cycle. Additional radiant heat was provided by 25W incandescent electric bulbs during daytime to reach a final day temperature of 35–37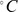. The locusts were fed daily with wheat seedlings and dry oats. Locusts, approaching the gregarious phase, were reared in 60-liter metal cages at a density of 100–160 animals per cage over many generations. All experiments were performed on nymphs of the final (Vth) nymphal-instar (3–4 cm in length and ∼0.5 cm in width). For a set of preliminary neurophysiological experiments, we used, in addition to the crowded animals, also nymphs approaching the solitarious phase. For this purpose, hatchlings from eggs laid by crowded locusts were isolated within 4 hours of hatching. Each newly hatched nymph was individually placed in a 1.5 liter metal cage and kept under isolation in conditions similar (all but the density) to the above.

### Behavioral setup

**Figure 1:**
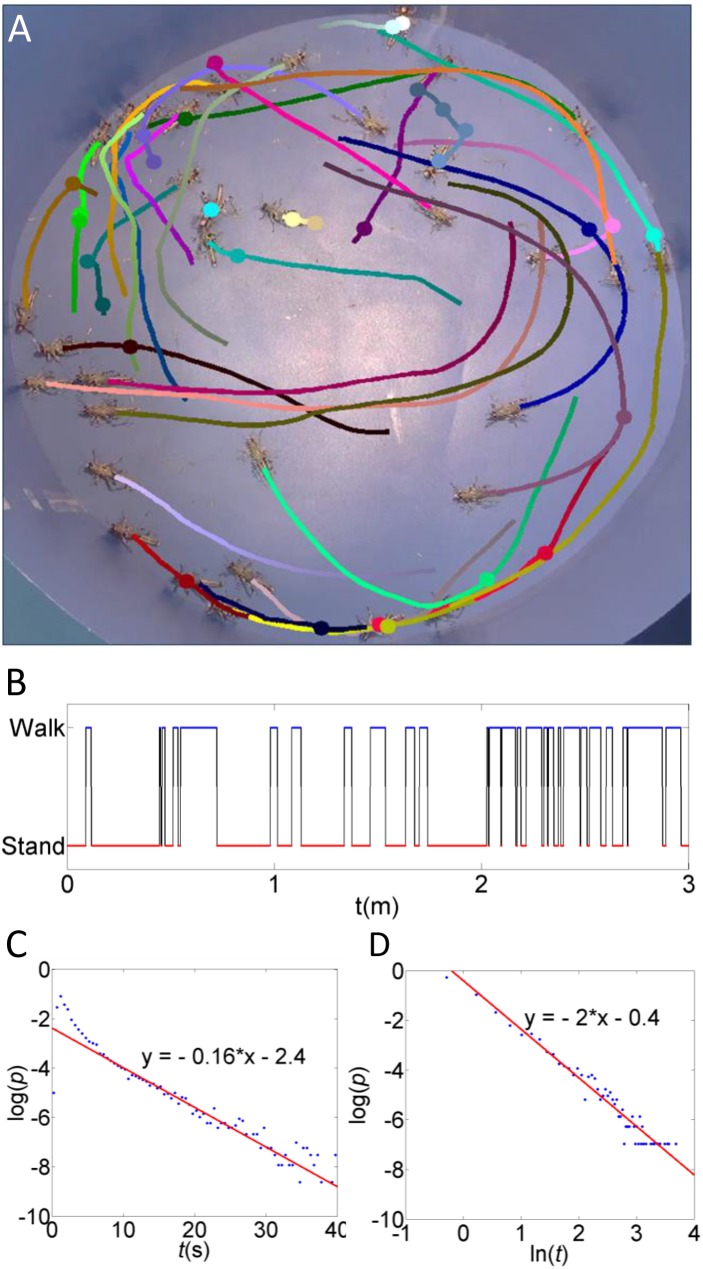
The motion of a single animal is characterized by an intermittent pause-and-go motion. Upon every walking initiation an animal makes a decision of whether or not to swarm based on tactile and visual stimuli. (A) A snapshot from an experiment showing the path of individual animals over 3 seconds. Filled circles show the location of pauses. (B) A typical sequence of pause-and-go transitions in a single animal. (C) The distribution of pause times shows a power-law decay. (D) The distribution of walk times is well approximated by an exponential distribution.

We conducted a series of experiments in which a group of crowded-reared last larval instar nymphs were freely moving in a homogenous circular arena with a diameter of 50 cm (see Figure 1A and Movie S1 for typical experiments). The arena was composed of a flat blue Perspex sheet limited by an outer flexible blue plastic circular wall (50 cm diameter×55 cm high; Figure S1). The lower 10 cm of the arena’s walls were thinly coated with glycerin to prevent nymphs from climbing. The arena was placed in our temperature controlled room (30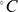) and lit from above by a single central 100W incandescent electric bulb. Nymphs were introduced to the arena in three different densities as detailed in Table 1, all high enough to allow the formation of synchronized movement [14], [15], [16]. Nymphs were continuously monitored and recorded using a Sony HDR-XR550E digital camera with a 25 fps rate for later off-line analysis ( Table 1 also presents duration of the experiments). Using a novel, custom-designed continuous multiple-target tracking method (see Multi-target tracking in SI, Figure S2), we simultaneously tracked the movement of all individuals with high spatial and temporal resolution (full HD allowed a detailed analysis of the behavior of each individual. Tracking data is available on line at http://u.math.biu.ac.il/~arielg/researchPages/PLoS1intermittentMotionData.html.

**Table 1:** Locust density and duration of the experimental trials.

Trial number	Number of nymphs	Density (locusts/m^2^)	Duration of recording (min)
**1**	34	173	60
**2**	38	193	180
**3**	54	275	130

### Analysis of behavior

Several behavioral events were defined as follows:

#### Pause or Go

To eliminate noise, an animal was considered to be moving if its speed was above a threshold for more than 10 consecutive frames. An animal was considered as pausing (or stopping) if its speed was below the same threshold for more than 20 frames. See figure S3 for the distribution of speeds in experiments.

#### Walking initiation

Walking initiation events were defined as transitions from pausing to movement periods. Such events were divided into those that occurred while the locust was being touched by one or more other nymphs and those that occurred without any tactile stimuli.

Next we averaged the number of walking locusts within a radius of 5 cm of the walking initiating nymph. This averaging was done over 300 consecutive frames (corresponding to 12 seconds), 150 before walking initiation and 150 after. The average change in the number of moving individuals surrounding a locust right before it started moving was the key visual stimulus we identified and utilized.

#### Walking direction

In order to determine the direction in which animals walk, we first observed that nymphs rarely changed their direction while walking - we observed but a few occurrences over several hours of experiments. Thus, we concentrated on the initial walking direction upon walking initiation. We compared three directions: head direction (measured at 5 frames prior to the event), initial walking direction (measured 10 frames past walking initiation), and the average direction of the active crowd (the order parameter, see below).

### The order parameter

To quantify order and synchronization in the system, we defined the instantaneous order parameter as the average velocity of walking animals in the angular direction. More precisely,
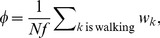
(1)Where 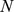 is the number of animals, 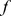 is the fraction of walking animals and 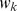 is the speed of the *k*’th animal in the angular direction: 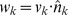, where 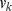
are animal velocities and 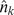 are normalized vectors pointing in the counter-clock-wise angular direction. When no animals are moving, the system does not have a preferred direction and 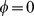. Thus, 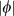 is a measure of the level of coordination within the swarm. The sign of the order parameter indicates the direction of the swarm: Positive 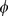 refers to counter-clock-wise (CCW) motion, while negative 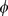 indicates clock-wise (CW) motion. Note that 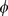 is different than the order parameter used by many authors since here it depends only on walking animals (See also [22]).

### Modeling and Simulation

In order to test the predictions derived from the behavioral experiments, two types of self-propelled particles models were simulated, a detailed model and a simplified model.

#### Detailed Model

The first model was a detailed approximate version of the actual animal-animal interaction observed experimentally. In this model, 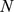 particles representing individual animals are distributed randomly on a circle with radius 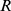, modeling an annulus in which locusts effectively move. Each particle heads either CW or CCW and can either stand or move at a constant speed. In this respect, the model is similar in spirit to traffic cell models and is also related to the a-synchronous model suggested by Bode et al. [23]. See the SI for a precise mathematical statement of the model.

The dynamics is determined by specifying the probability of a particle to start or stop moving and to change its head direction (turn). To be more specific, the model is defined by the particles’ (individuals’) probability to pause or start moving, and turn.

Pausing: A moving particle can pause due to two reasons, assumed to be independent: spontaneously, or following collisions with other particles.

Walking initiation: Standing particles can start moving due to three reasons, assumed to be independent: spontaneously, following tactile stimulation (collisions with other particles) or following visual stimulation (the average distance to conspecifics at the front is decreasing or the average distance at the back is increasing).

Turning: A particle that is starting to walk has a given probability of turning, which depends on its orientation relative to the order parameter. As observed in the experiments, the probability of turning depends on whether a particle is oriented with or against the crowd.

Table 2 details all model parameters and their default values. Other similar choices of simulation rules have been considered. For example, the probability of animals to start walking spontaneously may be drawn from the empirical distribution reported for isolated locusts in [19]. Simulations suggest that results are not sensitive to the precise expressions used.

**Table 2:** Parameters in the detailed model.

Parameter	Description	Default value
*N*	Number of particles	34
*R*	Arena radius	25
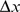	Grid spacing. Determines average velocity.	0.15
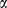	Probability to turn at 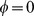	0.062
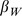	Slope of probability to turn with the direction of the crowd as a function of 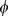	−0.021
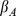	Slope of probability to turn against the direction of the crowd as a function of 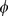	−0.055
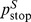	The probability (per time step) to stop spontaneously	0.0082
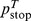	The probability (per time step) to stop when in the same location as another particle.	0.51
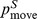	The probability (per time step) to start moving spontaneously	0.015
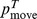	The probability (per time step) to start moving when in the same location as another particle	0.36
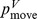	The probability (per time step) to start moving when the visual stimulus is on	0.04
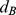	Threshold for the visual stimulus at the back of the animal	2
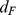	Threshold for the visual stimulus at the front of the animal	2
*r*	Interaction distance	5

In the detailed model all parameters are drawn from our analysis of the experiments. Given that the size of the experimental arena is comparable to the observed interaction distance between individuals (Figure S4), particles in the detailed model align with a global order parameter that includes all moving particles. As explained in the results section, we find that the probability of turning depends on 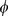. For simplicity, we assume a linear dependence,
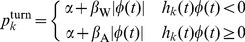
(2)Where 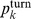 denotes the probability that particle 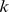 makes a U-turn, 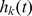 is the head orientation (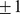) of particle 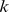 and 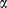, 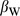 and 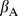 are parameters.

#### Simplified model

The second model was a simplified version that included a few key features of the actual locust dynamics, which we found to be essential for describing the macroscopic properties of the swarm. In addition, the simplified model considers larger swarms. For this reason, all interactions between particles are taken to be local. While the general setup of 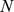 particles moving intermittently on a one dimensional circle is the same as in the detailed model, the interaction between particles is modified as follows (See the SI for a precise mathematical statement of the model).

Pausing: Spontaneous only with a fixed probability.

Walking initiation: Either spontaneous or locally triggered. We define an increased probability for starting to walk whenever the number of moving particles is higher than a given threshold.

Turning: the probability of turning depends on a local version of the order parameter 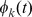, which is the average head direction of all moving animals that are located up to a fixed threshold distance 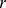 from animal 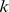. For simplicity, we assume a linear dependence with a minimum,



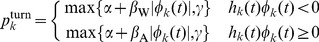
(3)


Table 3 details all model parameters and their default values.

**Table 3:** Parameters in the simplified model.

Parameter	Description	Default value
*N*	Number of particles	100
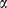	Probability to turn at 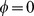	0.06
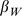	Slope of probability to turn with the direction of the crowd as a function of 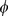	−0.05
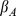	Slope of probability to turn against the direction of the crowd as a function of 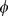	−0.08
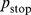	The probability (per time step) to stop (only spontaneously)	0.03
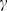	Minimal turning probability	0.003
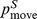	The probability (per time step) to start moving spontaneously	0.004
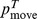	The probability (per time step) to start moving due to stimulus	0.05
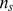	Threshold for the stimulus	2
*r*	Interaction distance	5

### Electrophysiology

Details of the dissection, DCMD nerve recording, visual stimulation and data analysis are provided in the Supplementary Material in File S1. In short, fifth-instar locust nymphs were anaesthetized and their legs removed (n = 8; 4, gregarious and 4 solitarious phase). Extracellular recordings of the DCMD spikes were made with silver hook electrodes from the neck connectives (DCMD action potentials can be faithfully identified by their large amplitude and characteristic response to visual stimuli). Computer generated visual stimuli were presented via two computer monitors in the front and back of the animal. Each animal was presented with four different visual stimuli (30 repeats each): single, or multiple simultaneous cues, approaching from the back, or receding in the front. Action potential times (relative to maximal object size), number and frequency were comparatively calculated.

## Results

### Intermittent motion

Most importantly, we found that desert locust nymphs in a crowd present a walking pattern of intermittently switching between standing and walking (various colored lines and circles in Figure 1A). Figure 1B shows a typical sequence of pause-and-go periods. The distribution of spontaneous pause times (in the absence of stimulus) could not be obtained directly from the experimental data, since pause times were interrupted by the external stimuli. In order to estimate the mean spontaneous pause time, each pause segment was labeled according to the event it ended with – tactile, visual or none. The first two were treated as right-censored events. A right-censored maximum likelihood estimate yielded an average spontaneous pause time of 2.3 seconds. Similarly, walks were terminated either spontaneously or due to tactile stimuli, considered as censoring. Due to the shape of the distribution of walking times we modeled the spontaneous walking time as a sum of two independent exponentially distributed random variables with different averages. A right-censored maximum likelihood estimate yielded 0.16 and 4.06 seconds.

Walk durations were well approximated by independent exponential random variables (Figure 1C). This observation indicates that animals terminate walks at random and the process has no memory (a Markov process). In contrast, pause times (Figure 1D) have a fat-tailed distribution that seems to decay with a power law scaling of approximately 2 (compare with Bazazi et al. [17], who found for single locusts a scaling factor of 1.67). The power law distribution suggests underlying information processing with memory, supporting our hypothesis that decision-making is involved during pauses. It is important to note that the intermittent walking does not seem to be an artifact of our experiments or the lab conditions, as it was also a dominant feature of individual locust hoppers in the recently observed naturally occurring marching bands observed in Israel’s Negev desert (e.g. Movies S2 and S3).

**Figure 2:**
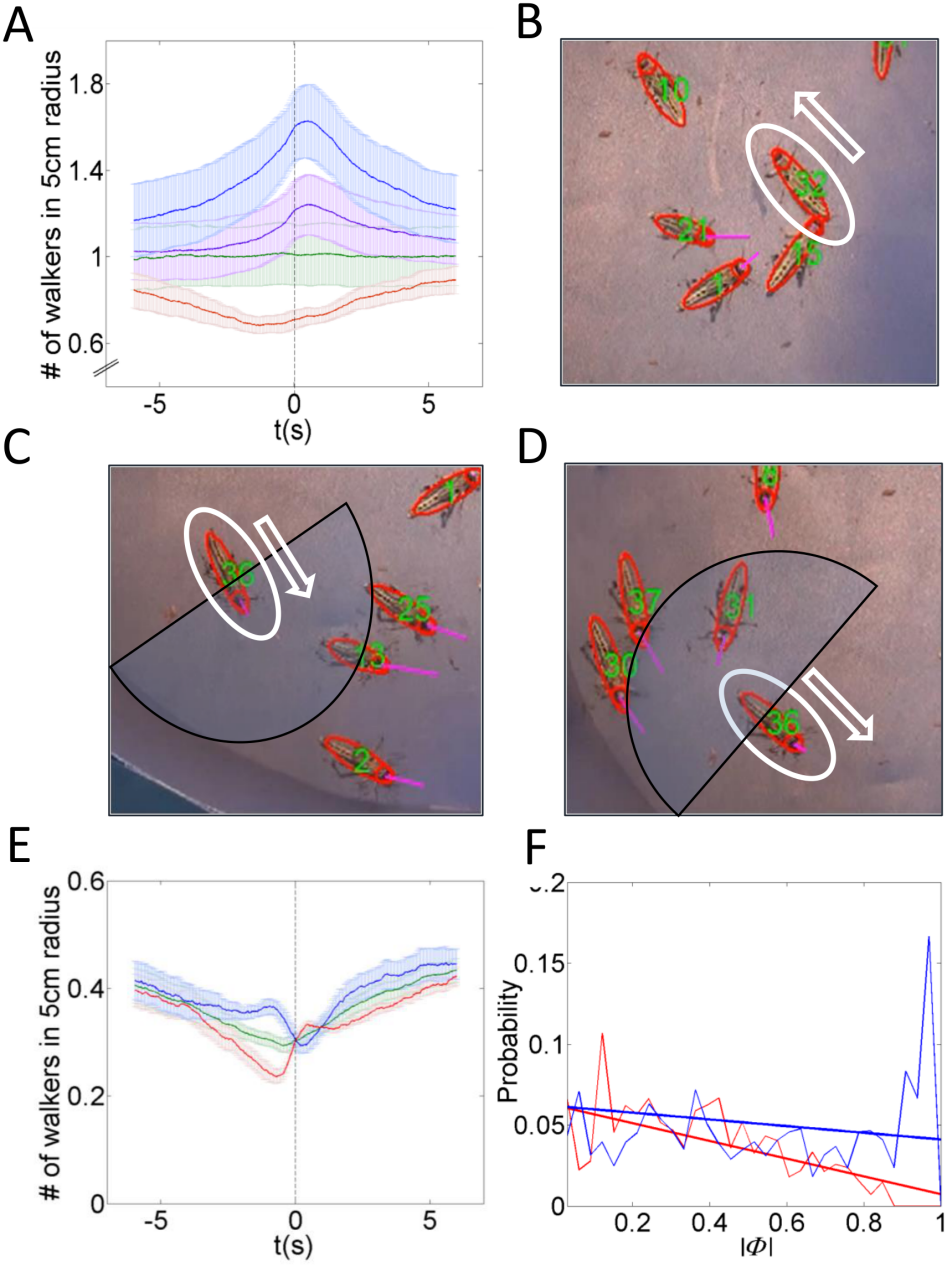
Experimental results. (A) The average number of animals walking within 5cm of to an individual that is starting to walk. The vertical line shows the time of walking initiation. Green line: random frames and animals. Purple line: all walking initiation events. Blue line: tactile stimuli-related events. Red line: non-tactile events. (B–D) Zoom-in showing the interaction between conspecifics. (B) Tactile interaction (C) Visual stimulus from the front and (D) Visual stimulus from the rear. (E) The average number of walking animals before and after walking initiation (time = 0). Colors indicate front (red), back (blue) and both (green). (F) The probability of changing orientation (U-turn) when resuming movement is a function of the order parameter. As 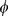 increases, the probability of turning in the direction the crowd moves (blue) becomes larger than the probability of turning against it (red). As expected, fluctuation at small 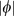 are large since the system spends a relatively short amount of time in this state. The line shows a linear least squares fit as a guide to the eye.

Next, we looked for the mechanisms involved in the decision of a standing locust to initiate or resume walking (rejoin the marching crowd). We explored various potential triggers by scanning all walking initiation events in our experiments (all in all some 70,000 events). The most consistent factor was found to be an increase in the average number of walking animals in close proximity of the standing individual (compare the purple line in Figure 2A to the green line denoting randomly selected frames). Further investigation revealed that in many cases walking initiation was preceded by the standing individual being touched (or “bumped”) by a walking animal (Figure 2B). Indeed 54% of walking initiation events could be explained by tactile stimuli. The distribution of touch angles is depicted in Figure S5A. As expected, tactile-stimuli-related walking-initiation events were preceded by an increase in the average number of walking animals in close proximity of the touched locust (blue line in Figure 2A). This phenomenon may well be a result of the physical constraints of the experimental arena, as “bumping” was rarely seen in the wild marching bands (movies S4 and S5).

Interestingly, when focusing on all walking initiation events that did not involve tactile stimuli (46%), and again looking at the average number of walking animals in the vicinity of the standing individual, data was still markedly different from that generated by random (compare the red and green lines in Figure 2A), suggesting an additional mechanism beyond tactile stimuli, that is responsible for walking initiation (or resumption of walking) by a standing locust. This additional mechanism translated to the standing locust seeing a decrease in the number of moving animals in its vicinity. To further investigate the visual stimulus that potentially induces walking initiation we separately examined the optical flow in the front versus the back part of the walking-initiating animal’s visual field (Figure 2C, D). We found that, on average, right before an animal (which is not being touched) starts moving it senses a reduction in the number of moving individuals in front of it, or an increase in the number of moving individuals behind it (Figure 2E). Assuming different, 1–13 cm, radius spheres around the standing locust, we tried to determine the effective range of visual interactions between the nymphs. To do so, control curves were calculated for each radius using 10,000 cases of random frames and random animals and were deducted from the walking initiation curves (Figure S4). We then tested whether curves of subsequent radii differ in their distribution, using a set of two-samples Kolmogorov-Smirnov tests. We found that for both the front and back visual fields, beyond a radius of 9 cm, the curves are not statistically different (Table S1 in File S1).

The described interactions may be reminiscent of the escape-and-pursuit behavior mentioned above. They are consistent with the results of the visual occlusion experiments reported in [18], as, similarly, we suggest a strong dependence on visual inputs. However, the behavior we identified is not directed at the individual locust. Moreover, locust tend to walk in transitional-parallel paths (Figure 1A, movies S1, S2 and S3). They regularly walk in the direction their body axis is pointing (Figure S5B shows the distribution of angles between the head direction before walking and the velocity direction after, i.e., the turning angle), and rarely change their direction, nor turn back (make a U-turn) while walking.

### Turning and the build-up of synchronization

We observed that both the decision to start walking and specifically to walk “with the crowd” are closely correlated with the order in the system (see Materials and Methods for definition of the order parameter).

We tested the evolution of the overall fraction of “walkers” and the order parameter (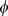) during the first 15 minutes in our experiments, see movies S4 and S5. The build-up and persistence of order is depicted in Figure S6. The size of 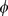 and the fraction of walkers were correlated with a correlation coefficient of 0.38, showing that the tendency of animals to join their conspecific increased with the number of walkers. As explained above, we observed that nymphs rarely changed their direction while walking and thus we concentrated on the turning event during movement initiation. We find that turning depends on the level of order in the system. Figure 2F shows the probability of changing orientation (from CW to CCW or the other way around) as a function of the order parameter. The probability of animals to start walking in the same direction as the crowd (blue line in Figure 2F), becomes larger than the probability of walking against it (Red line in Figure 2F) as 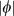 increases. This is a key observation, which is essential to the emergence of order and synchronization within the marching band.

Based on these findings, the mechanism underlying the build-up of synchronization can be described as a positive feed-back on the fraction of walkers: an increase in *f* increases the frequency of walking stimulus and therefore tends to increase the number of walkers. Since all animals occasionally stop, *f* fluctuates around a meta-stable equilibrium value. The correlation between *f* and 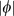 implies that qualitatively the order parameter behaves similarly. The main difference is that, due to symmetry, 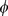 has two meta-stable equilibrium points related to CW or CCW directions, 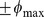.

**Figure 3:**
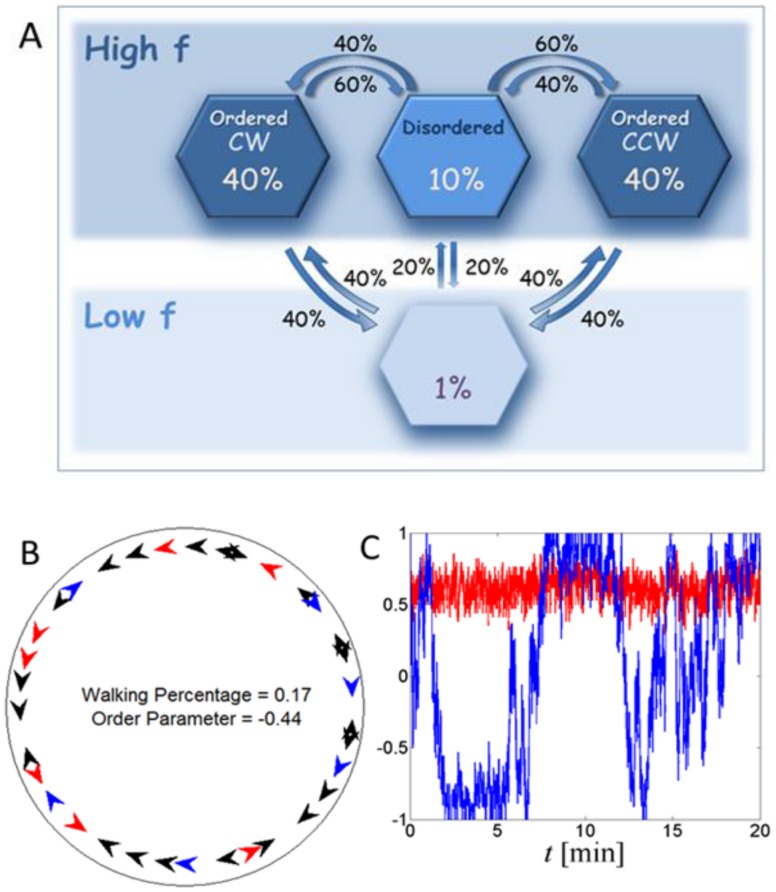
Detailed model results. (A) The dynamics of the system can be approximated by a coarse-grained continuous-time Markov chains with four states: A relatively static state in which most of the animals are standing, and three active states in which most of the animals are walking. The three active states can be classified according to the order parameter and correspond to one disordered and two ordered movement patterns. Numbers show the relative time the system spends in each state and the transition rates as obtained in simulations of the detailed model. (B) A snapshot from simulation showing standing (black) and moving (CW-blue, CCW-red) agents. (C) The time evolution of the fraction of walkers (red) and order parameter (blue) in a typical simulation.

The positive feed-back mechanism described above can be disrupted due to large fluctuations, which may be considered as rare events. With a large enough number of walkers, the bias in the direction of movement implies that the sum 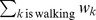 in Eq. (1) is proportional to the number of walkers 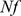, and 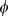 approaches one of its meta-stable values. However, when the number of walking animals is small, fluctuations dominate and 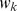 are practically random. This implies that 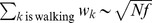 and 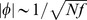. Therefore, an increase in 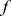 results in a decrease in 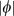. We conclude that under the mechanisms described above, the disordered state 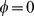 should also be meta-stable. This is qualitatively different from the prediction of previous locust models in which, while the system is in an active phase (high 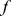 ), disorder is an intermittent, unstable state of the system [16], [21], [22], [23]. More precisely, we identify meta-stable states, characterized by regions in the 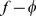 plane in which the dynamics is confined to for relatively long times. Transitions between such regions should be rare and the time between them distributed exponentially. Four meta-stable states are depicted in Figure 3A: a relatively static state in which most of the animals are standing, and three active states in which most of the animals are moving. The three active states can be classified according to the order parameter and correspond to one disordered and two ordered movement patterns - CW and CCW. Even though the time that the system spends in the static state is short, its impact on the dynamics is pivotal, as a large percentage of transitions are to and from this state (see also [22]). Intuitively, it is easier for the system to change its orientation by first reducing the number of walkers, and thus locally increasing the influence of fluctuations.

### Order and disorder under pause-and-go movement

**Figure 4:**
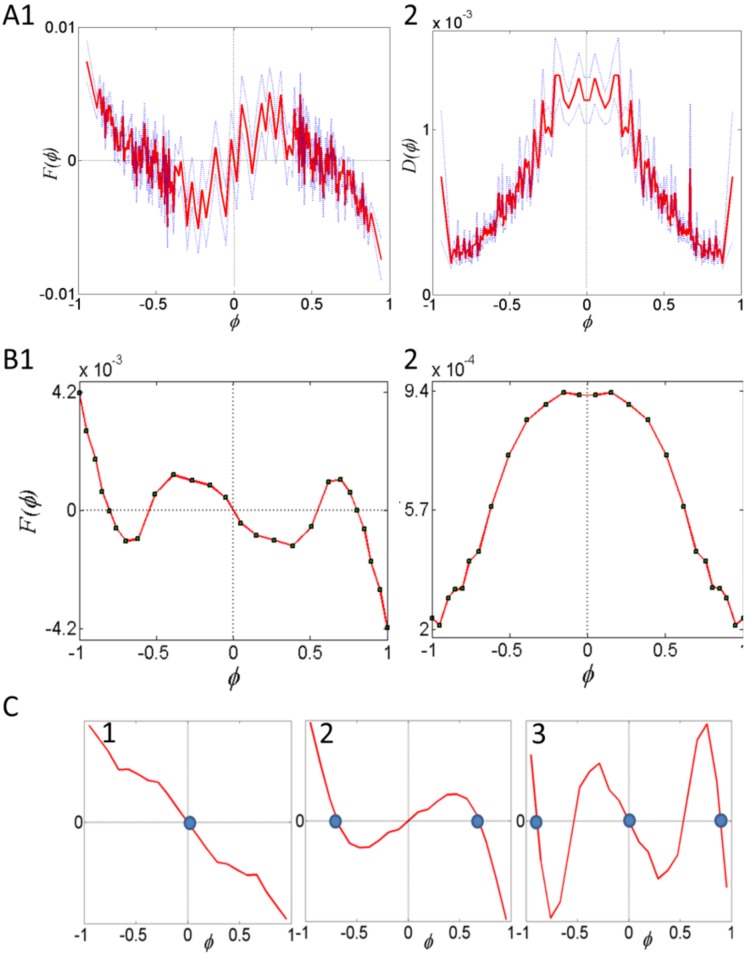
Effective dynamics of the order parameter in experiments. (A) Experiments. 1. The effective drift. The zeros of 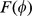, corresponding to fixed points, cannot be observed due to a large statistical error. 2. The effective diffusion shows a clear peak at small values of 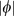. Compare with Yates et al. [21]. The blue dotted line indicates the statistical error. (B) Detailed model. 1. The effective drift shows three meta-stable states. 2. The effective diffusion. (C) Simplified model – the predicted behavior of large swarms. The figure shows the effective drift function as obtained in a simplified model with several hundred particles. By changing the probability of turning, the admissible meta-stable states of the system are either 1. Only disordered, 2. Only ordered 3. Both ordered and disordered. Hence, social interactions determine the macroscopic dynamics of the system. Statistical errors in (B) and (C) are of the order of the size of the symbols.

To further quantify this behavior, we describe the effective dynamics of 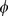 as an approximate diffusion process [15], [23] of the form.
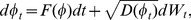
(4)where 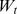 is the Weiner process (Brownian motion). In (4), 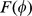 is the effective drift function that describes the dynamics of the average 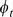. Indeed, denoting the average order parameter at time 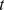 by 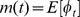, the time evolution of 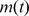 is given by the ordinary differential equation 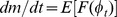. The effective diffusion function 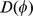 describes, loosely speaking, the amount of noise that the system is subject to. The functions 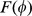 and 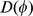 can be approximated from the dynamics as



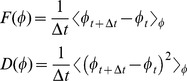
where brackets denote averaging over all frames in which the order parameter is in some range of 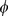 and 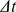 is the time between frames. Figure 4A depicts 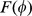 and 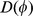 obtained from experiments. As expected, 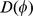 has a maximum at 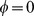. This is a small numbers effect which is a direct consequence of intermittent movement behavior (compare with [21], [23], [26]. The meta-stable states of the system are characterized by stable zeros of the drift function 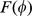. Stable means that the system will return to the zero point following a small perturbation. In other words, close to the stable point, if 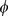 increases, 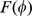 is negative, which means that, on average, 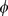 will decrease. On the other hand, if 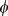 decreases, 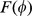 is positive, and 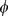 will increase back to the fixed point. Other zeros of 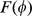 are unstable to small perturbations. Unfortunately, the data for 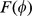 are too noisy to conclude the existence of a meta-stable disordered state at 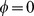 (A meta-stable disordered state is weakly discernible in [21]; their figure 2B).

We suggest that the fundamental process, which governs the dynamics of the swarm, is that of visual-flow-based repeated decisions that each individual animal makes regarding when to pause or start walking, and in what direction. Moreover, the individual pause-and-go strategy has profound implications for the macroscopic behavior of the swarm, as it facilitates the emergence of collective order. We sought to test these hypotheses and predictions by a mathematical model.

### Modeling and Simulation

The dynamics obtained in our experiments were simulated using two types of self-propelled particles models. The first, a detailed model, which includes an approximated version of the actual animal-animal interaction observed experimentally, and the second a simplified model.

#### The detailed model

This model was used to verify the consistency of our description of the dynamics as deduced from the experiments. In addition, simulations allow an extensive investigation of the contributions of the different model constituents and ample sampling. See Figure 3B and movie S6 for a snapshot from a simulation.

Figure 3C shows the evolution of the fraction of walkers, 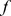, and the order parameter, 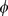, in a typical simulation. The distribution if 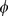, 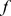 and the correlation between them is depicted in figure S7. As described above, we identify four meta-stable states, corresponding to a static state in which most of the animals are standing, and three active states corresponding to one disordered and two ordered movement patterns - CW and CCW. Accordingly, the dynamics of the model can be well approximated by a four-state continuous time Markov chain. The probability of being in each state as well as the transition times is depicted in Figure 3A. Waiting times between transitions are approximately exponential, in agreement with the Markov assumption (Figure S8A). Figure 4B shows the effective drift and diffusion functions as obtained from the detailed model, see also Figure S9. Compared with the experimental result, Figure 4A, the general trend is similar. However, the experimental data is too noisy for a precise comparison. As predicted, we find that in simulations, 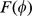 has five roots, corresponding to the fixed-points of 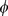. Two of these points are unstable, while three are stable – referring to one disordered and two ordered states. The sensitivity of this prediction to the model parameters is examined in the SI section and summarized in figures S10 and S11.

The shape of the effective diffusion coefficient 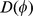 can be compared with two previous models: Yates et al. [21] and Bode et al. [23]. Yates et al. [21] suggest a model in which time is continuous. In simulations, time steps are taken synchronously, i.e., the positions and orientations of all particles are updated together. As a result, the shape of 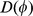 is flat. It does not show a peak around 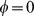 and the authors needed to introduce a non-trivial, ad-hoc 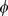 dependent, noise term in order to account for this observation. Bode et al. [23] show that changing simulations to an asynchronous scheme, in which particles are updated at random (Poisson distributed) times, qualitatively reproduces the correct form for 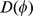 without any special assumptions on noise. In our model, random waiting times are analogous to the asynchronous scheme of Bode et al. [23]), which accounts for the observed diffusion. However, the model of Bode et al. does not show the positive feedback between the order parameter and the number of walkers and hence does not explain the meta-stable disordered state.

#### The simplified local model

The detailed model described above captures the system dynamics as observed in experiments quantitatively. However, due to the relatively large number of model parameters (see Table 2), it is difficult to understand the importance of each parameter and its effect on the swarm dynamics. Accordingly, in order to identify the key principles leading to the kinetic order-disorder transition as predicted by our experiments with marching locust bands, the detailed model described above was simplified, stripping it of many of the experimental details. As a result, we can no longer expect a quantitative agreement between simulations and experiments. In the simplified model, we assume that whenever the number of moving particles around a standing individual is above a given threshold, the standing individual has an increased probability to start walking. In addition, all interactions between particles are taken to be local. This implies that the instantaneous description of a particle does not depend on the order parameter of the entire system, but only on a local version that considers a few close neighbors (See the Materials and Methods and SI sections for details).

Once again, the dynamics fits the 4-state CTMC picture depicted in figure 3A with slightly different occupancy frequencies, transition probabilities (Table S2 in File S1) and waiting times (Figure S8B). Interestingly, the time the system spends in the small 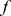 regime decreases, but its effect on the dynamics increases as most of the transitions between meta-stable states happen through it. Simulations with more particles yield similar results. In fact, the four-state CTMC approximation becomes more even accurate with larger *N*.

As explained earlier, the probability of an animal to change direction depends on the local order around it. In other words, it is the “social” interaction that determines the probability of individuals to join the crowd or walk against it. Indeed, Figure 4C shows that by changing this probability, specifically 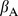 and 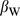 in Eq. 3, the available meta-stable states can change. With a lower probability of turning against the direction of the crowd, only a single disordered state exists; while with a higher probability, only ordered states exist. However, within the range of parameters corresponding to the experimental conditions, all three states are accessible. Thus, the social interaction between individuals determines the available macroscopic states of the system and the proportion of time the system “gets-stuck” in each state before transitioning to a different one. The sensitivity of these observations to some of the other model parameters is examined in the SI section and summarized in figure S12.

A related observation was presented by Bhattacharya and Viscek [27] who modeled collective decision in lading bird flocks. In their model, each bird has an internal state that describes a bird’s tendency to land, similar to stopping in locusts. This state is influenced by neighboring birds, which results in a sharp transition to a stationary state in which the entire flock lands within a short time. However, in their model, stopping is not reversible and a bird that landed does not take off again.

### Neurophysiological evidences for the role of visual cues in locust collective movement

**Figure 5:**
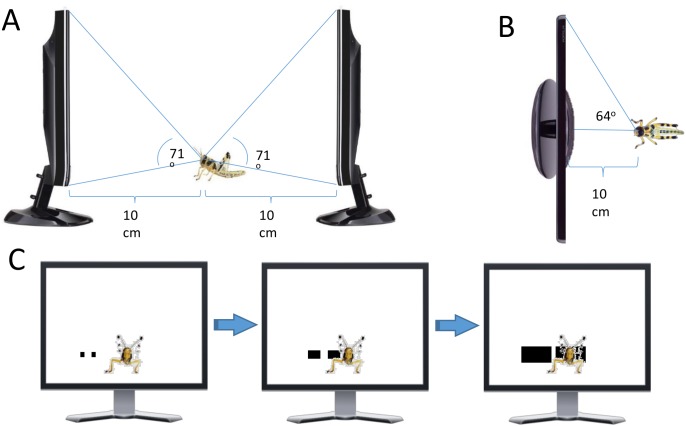
Experimental setup and visual stimuli used for DCMD recording. Locust was mounted in between two computer monitors (1366×768 pixels; A, side view; B, top view showing only front monitor). Details of the relative position of the animal in relation to the screens’ surface are shown, as well as an example of one stimulus type (two objects approaching in the back visual field) as seen on the back monitor (C, looking at the animal from the front).

We sought to offer preliminary evidence supporting the role of visual cues in the decision of a locust to join the marching crowd, by first demonstrating that visual stimuli, related to locust marching and animal interactions within a marching swarm evoke consistent neuronal responses. We presented gregarious locusts at the Vth nymphal stage with two basic visual stimuli (Figure 5), based on those observed in our experimental arena and supported by the model: either objects approaching in the rear visual field, or receding in the front visual field (Fig. 2C, D). Simultaneously, we recorded extracellularly the locusts’ DCMD neurons. As mentioned earlier, these motion-sensitive neurons were very thoroughly studied in adult locusts in relation to looming objects during flight, either fast approaching predators or flying locusts in a collision course (e.g. [28], [29], [30]. It is, however, important to note that here we used very different stimulus parameters as we simulate walking nymphs (much slower moving, significantly lower *l*/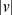 ratio; see Materials and Methods).

**Figure 6:**
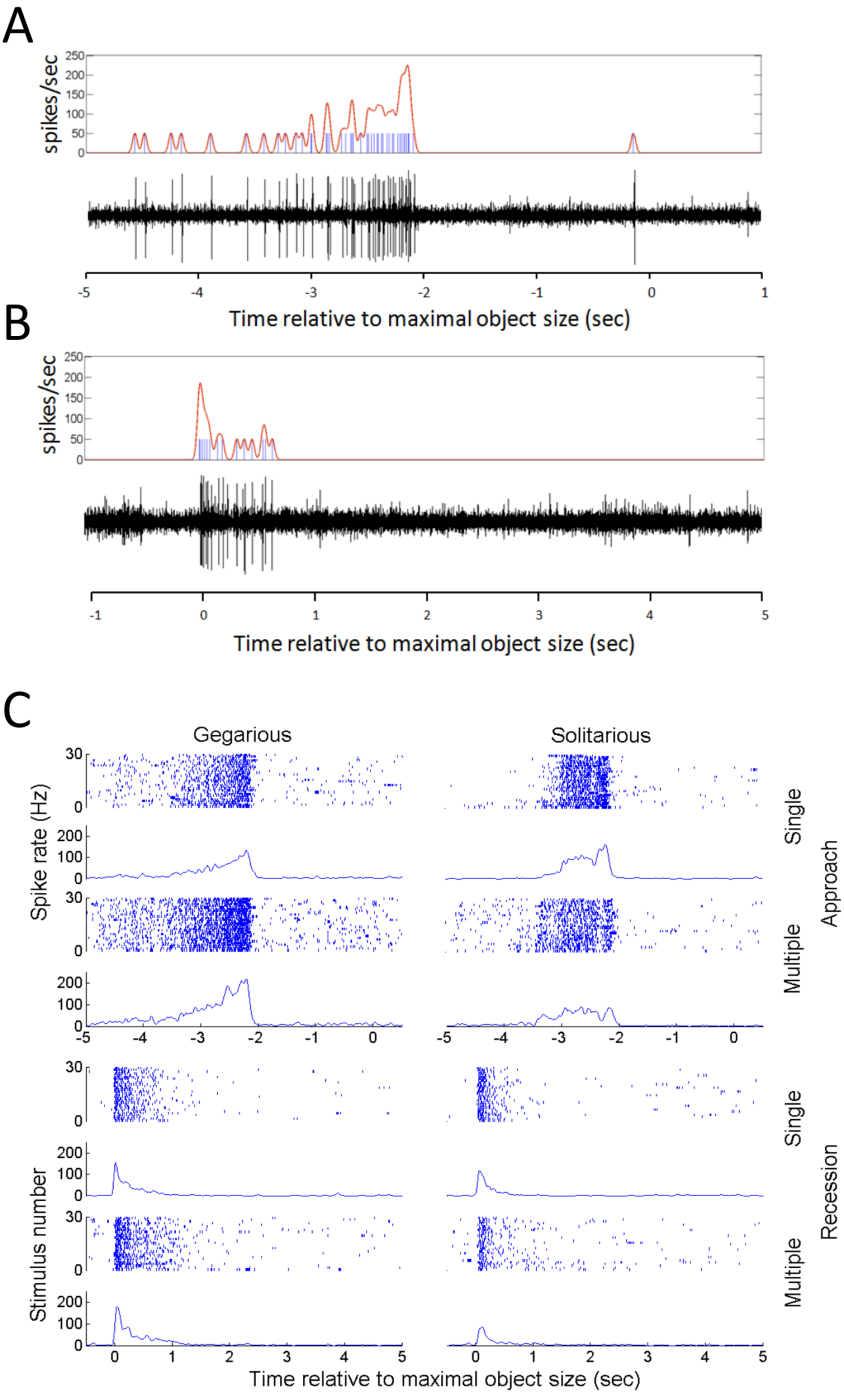
Typical response of the DCMD to approaching (A) and receding (B) stimuli. DCMD spike occurrence times (blue) were extracted from the extracellular recordings (black). Individual raster trials were then smoothed with a 20 ms Gaussian window and an evaluation of the instantaneous firing rate (red) was calculated by normalizing the resulting waveform so that its integral equals the number of spikes in the trial. (C) Firing patterns of DCMD in a solitarious and a gregarious animal, in response to the four types of visual stimuli. Each raster plot includes the response of DCMD to 30 sequential stimulations of the same kind, with the first at the bottom of the stack. Mean instantaneous firing rate across trials is shown in the histogram below each raster plot. While the response of DCMD to a single approach and recession bear similarity between the two phases, gregarious nymphs show higher numbers of spikes in response to multiple approach and recession than solitarious animals.

In the absence of visual stimulation, the DCMD fired at a low spontaneous rate of 0.696±0.863 spikes/sec (*n* = 4). All nymphs showed similar responses to an approaching object, which resembled typical DCMD looming responses (Figure 6A). Firing started early during the approach phase and its rate increased gradually as the object grew larger. Peak firing rate preceded the approaching object’s maximal size by approximately 2 sec. The DCMD response to receding objects was also consistent with previous observations [31], [32], with a peak firing rate aligned with the recessing object’s maximal size (Figure 6B). The firing rate then gradually decreased as the object grew smaller.

An approaching object generated a significantly higher number of elicited spikes compared to a receding one (37.5±9 spikes and 19.8±7 spikes respectively, repeated measures ANOVA, F_1,6_ = 8.96, *p* = 0.024). We found no effect for the type of stimulus (i.e. approaching vs. receding) on peak firing rate (F_1,6_ = 0.29, *p* = 0.61). Each stimulus was presented 30 times consecutively (see SI), and data revealed a strong effect of trial number on the number of elicited spikes (F_29,174_ = 3.1, *p*<0.001), i.e. habituation of DCMD response, which will be described in details below.

Based on our behavioral observations and model, we expected multiple simultaneous visual cues to induce a stronger visual response compared to single cues. Each of the nymphs was therefore presented with two additional kinds of visual stimuli: two objects approaching in the back visual field (Figure 5) and two objects receding in the front visual field. The DCMD responses between all four kinds of stimuli (single or multiple stimuli, recession or approaching) were compared, over all 30 trials, including peak firing rate and number of spikes. We found that visual stimulus type affected mean peak firing rate (F_3,12_ = 4.28, *p* = 0.028; single recession 164±40 spikes/sec, single approach 177±30 spikes/sec, multiple recession 224±31 spikes/sec, multiple approach 239±35 spikes/sec), with a significantly higher peak firing rate induced by multiple visual cues compared to single ones (*p* = 0.0045). A similar effect was found when analyzing the mean number of spikes (F_3,12_ = 16.5, *p*<0.001; single recession 19.8±7 spikes, single approach 38±9 spikes, multiple recession 30.5±6.8 spikes, multiple approach 66±14 spikes; *p* = 0.0015 for multiple versus single cues). Both mean peak firing rate and mean number of elicited spikes were affected by trial number (F_29,348_ = 1.5, *p* = 0.05 and F_29,348_ = 2.8, *p*<0.001, respectively). These habituation effects are further discussed below.

Finally, we examined our findings in light of locust phase polyphenism. In accordance with [33], we hypothesized that while habituated, the DCMD of gregarious animals will respond more adaptively to visual stimuli of different intensities, compared to solitarious animals. More specifically, in response to multiple visual cues, we expected a more pronounced response in gregarious animals compared to solitarious ones.

Solitarious animals were presented with the same four types of visual stimuli, including single and multiple approaching and receding objects, and their DCMD response was recorded and analyzed as above. In the absence of visual stimulation, we observed no phase-dependent differences in the DCMD activity (solitarious: 0.7±0.86 spikes/sec; gregarious: 0.9±1.0 spikes/sec; Mann-Whitney U = 7, *p* = 0.88, 6 df). Figure 6B presents a typical DCMD response of one gregarious and one solitarious locust to consecutive repetitions of the four types of stimuli used (single or multiple, approaching or receding). While the first visual stimulus induced strong responses in both phases (47±23 spikes for solitarious and 46±21 spikes for gregarious locusts), by the last trial, solitarious animals showed a much weaker response than gregarious ones (25.1±15.4 spikes and 37±23 spikes respectively; Figure S13). This represents strong habituation, to a level of 53% of the initial response, in solitarious nymphs, compared to weaker habituation in gregarious animals, to a level of 79% of the initial response.

Following [33], datasets of consecutive repeated recordings were fitted with a single exponential of the form y = y_0_+ae^−bx^ (see SI and Figure S14). Analysis showed that the two phases differ in their habituation fitted regression lines (F_2,22_ = 8.54, *p* = 0.0018). This effect was due to a difference in the gradient of the habituation curves (*p* = 0.0025) and not in their intercept (*p* = 0.18), suggesting a different intrinsic habituation rate for each of the two phases, as expected. While, habituation was also expressed in the effect of trial number on the DCMD peak firing rate (F_29,667_ = 2.319, *p*<0.001), the change in this parameter did not differ between the phases. Mean instantaneous firing rate across trials is also shown in figure 6B. Most interestingly, and consistent with our expectations, was a phase-related difference in the mean number of spikes induced in response to the movement of single versus multiple objects. Overall the gregarious nymphs showed a higher number of spikes than solitarious animals in response to multiple approaching stimuli as well as to receding ones, both kinds of stimuli relevant to a marching swarm.

## Discussion

### Locust phase polyphenism and swarming

It is important to address the consistency of our new description of locust behavior with the wealth of previous knowledge. Ever since Uvarov suggested his theory of locust phases [34], locust phase polymorphism has been established as one of the most striking examples of environmentally-induced phenotypic plasticity. Ample research has demonstrated that the gregarious-swarming and migrating phase differs from the solitary-sedentary one in a multitude of phenotypic traits, but first and foremost in its behavior [16], [35], and [36]. Gregarious *S. gregaria* have been characterized by a strong attraction to conspecifics, which translates to active aggregation behavior [37], [38]. They were also reported to be generally more active than solitarious locusts. The question remains as to whether these principal behavioral characteristics are sufficient to account for the locusts’ strong propensity to wander in huge bands of marching hoppers (or in flying swarms of adults). Specifically, are these characteristics also responsible for the striking coordinated behavior and synchronization seen in the marching bands?

Previous studies have suggested different models to account for what has seemed to be missing in our knowledge of locust behavior (e.g. a cannibalistic impulsion; [19], [20]. Here we suggest that the coordinated marching behavior of locust swarms is manifested by repeated decisions taken by individual animals to initiate or resume walking. Yet, it is actually the outcome of the fundamental locomotion-related behavioral characteristics of locusts in the gregarious phase. These recurring decisions can be explained simply by the high propensity for walking, supported by sensory stimuli conveyed by the optical flux in the vicinity of the animal (due to the behavior of others in the crowd).

### Visual stimuli and swarming behavior

There have been several reports presenting density-dependent differences in the processing of different sensory modalities between the locust phases (e.g. [33], [39], the former referring specifically to visual stimuli). We show for the first time that the DCMD neurons convey information relevant to the locust response to small, slow moving objects (such as other marching locusts). We used the DCMD response as a “proof of concept” and by no mean imply that this is the only or even major motion sensitive sensory pathway employed during marching and swarming. While other motion sensitive neurons have been described in locusts, a recent study by Simmons et al. [32] supports our choice to focus on the DCMD. In this study of larvae of the migratory locust, the authors report that from as early as hatching the larval DCMD neurons already respond selectively to objects approaching the locust and that they discriminate between stimulus approach speeds (tested speeds were 0.5–5 ms^−1^). Both approaching and receding stimuli were tested, and interestingly it was found that the response change with development: strong response to receding stimuli early in life, changes towards the adult stage into an improved response to objects approaching on a collision course. It should be noted that swarming and marching starts in the desert locust as early as a few days after hatching. Again, in accordance with our focus on the DCMD, Dick and Gray [40] suggest that this pathway is capable of responding uniquely to complex aspects of object motion, including translation and trajectory changes at different velocities. Matheson et al. [33] showed phase-related difference in the DCMD response of adults to flight-related looming objects, while in habituated state. It was suggested that tuning the DCMD responses to the speed and size of approaching objects may infer gregarious animal with specific responses to approaching locusts or approaching predators. Similarly, we suggest that during the larval stages gregarious locusts are almost constantly surrounded by moving nymphs. So while the swarm is on the move, their DCMD is in a continuously habituated state, yet still well-tuned to small, slow approaching or receding objects. This flow on the animals’ visual field is instrumental in the repeated decision taken to join the crowd, facilitating and coordinating the marching behavior of the swarm.

The difference in neural processing of visual stimuli between the solitarious and gregarious nymphs offers a simple and sufficient mechanism for the spontaneous emergence of large locust swarms in the latter. In solitarious nymphs visual stimuli are quickly habituated, and thus they are less inclined to walk (smaller 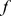), which corresponds to low order (smaller 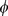). As a result, fluctuations are large and the system is in a disordered meta-stable state. On the other hand, gregarious nymphs have an increased probability to start walking (higher 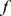), which is sufficient to push the swarm into a highly ordered state. Of course, this is just one aspect of the complex phenomena of locust swarming.

### Metastability and intermittent motion

Pause-and-go or, in general, intermittent motion, occurs in a wide variety of organisms [41], [42]. For example, it is well known that many types of fish swim in a burst-and-coast motion, possibly to optimize energy usage [43]. These discrete bursts of movement associated with rapid changes in speed and orientation parallel the repeated decisions sequence described here for locusts. Indeed, it has been recently shown that the dynamics of fish schools can be depicted using a diagram similar to Figure 3A consisting of stationary (low activity), ordered (polarized or milling), and disordered meta-stable states [4].

The similarities between fish and locusts suggest that the pause-and-go strategy may lead to a generally applicable dynamic pattern in which both disordered and ordered states are meta-stable. Transitions between ordered and disordered states are not necessarily caused by external or internal changes but are, rather, dynamic states of the system. In particular, there is no phase transition between order and disorder in the sense of statistical physics [1], [3]. The effect of the environment and other parameters, such as the animals’ concentration [15], diet [19] or behavioral phase [25], lies in changing the probability of the system to be in either one of the meta-stable states, and in changing the transition rates between the two states while in equilibrium. In the future, it would be interesting to compare the behavior of locusts with that of other organisms showing intermittent motion and look for a more general theoretical explanation.

## Supporting Information

Figure S1 The experimental setup: Several dozen locusts are placed in a plastic circular arena with a diameter of approximately 50 cm.
(TIF)

Figure S2 Video analysis algorithm. (A) A hand-labeled frame in which animal were painted red and some of the background was painted blue. (B) The RGB content of labeled pixels. The two clusters are separated using a support vector machine. (C) All pixels in each frame are classified as either animal (red) or background (blue). (D) Labeled regions that fit certain size properties. (E) Numbered objects. (F) Voronoi cells associated with individual animals.
(TIF)

Figure S3 Experimental results: The distribution of walking speeds.
(TIF)

Figure S4Experimental results. The optical flow in the front (A) and in the back (B) halves of the walking initiating animal’s visual field. Each curve shows the average number of walkers at time *t* from a walking initiation event within a given distance from the animal that is starting to walk. For example, to generate the purple curve we listed all walking initiation events. Suppose that during event 1, animal *k* start walking at time 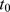. We counted the number of animals walking within a distance of 5 cm from animal *k* at time 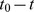 and then averaged over all walking initiation events. Other distances we calculated in a similar manner. While in the front, a walking initiation is preceded by a reduction in the number of moving nymphs, an increase in the same parameter is seen in the back. In both halves of the visual field the signal saturates at around 9 cm, suggesting that above this radius no further visual information regarding the animal’s surrounding is obtained.(TIF)

Figure S5 Experimental results: Angles. (A) A histogram showing the distribution of angles in which animals that started walking due to a tactile stimulus were touched. (B) The angle between the head direction 10 frames before walking and the velocity vector five frames after walking started.
(TIF)

Figure S6 Experimental results. The time-evolution of the order parameter (blue) and fraction of walking animals (red) in the three experiments described in Table 1. The two variables are correlated with an average correlation coefficient of 0.38.
(TIF)

Figure S7 Detailed model results: The distribution of the average order parameter (A), average fraction of moving particles (B), and the correlation between the two (C) in 1500 simulations with length corresponding to 30 minutes of experiment time.
(TIF)

Figure S8 The distribution of waiting times between transitions in the 4-states CTMC approximation. (A) The detailed model. (B) The simplified model.x: state 1, o: states 2 and 4, +: state 3. Solid, dashed and dotted curves are a maximal likelihood fit to an exponentially distributed random variable from states 1, 2+4 and 3, respectively.
(TIF)

Figure S9Detailed model results: Parameters for the diffusion equation describing the effective dynamics of the order parameter 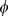, showing three meta-stable states. (A) The drift, *Fφ* with three stable roots. (B) The diffusion coefficient, *Dφ* has a maximum around zero. (C) The potential, *Uφ*, and (D) The probability density function, *fφ*.(TIF)

Figure S10Variation of parameters in the detailed model. Top row: number of particles 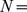
10 (blue), 15 (red), 34 (green-experimental value), 100 (black). Middle row: Probability to V* = *0.1 (blue), 0.2 (red), 0.3674 (green-experimental value), 0.4 (black), 0.5 (purple). Bottom T* = *0.02 (blue), 0.03 (red), 0.039 (green-experimental value), 0.05 (black), 0.06 (purple).(TIF)

Figure S11Variation of parameters in the detailed model. Top row: Probability to stop moving* = *0.3 (blue), 0.4 (red), 0.5083 (green-experimental value), 0.6 (black), 0.7 (purple). Middle row: 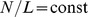. 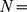
10 (blue), 15 (red), 34 (green-experimental value), 50 (black),70 (purple). Bottom row: Interaction radius *r = *3 (blue), 4 (red), 5 (green-experimental value), 6 (black), 7 (purple). As 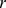 changes the average number of interacting neighbors, this is also equivalent to changing the interaction thresholds 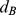 and 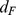.(TIF)

Figure S12Variation of parameters in the simplified model. Left column: effective drift, middle column: effective diffusion and right columns: effective potential. Top row: dependence on 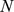, Middle row: dependence on 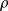 and bottom row: dependence on 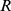.(TIF)

Figure S13 Phase related differences in DCMD response. (A) The habituation observed in the elicited number of spikes was more substantial in solitarious nymphs than in gregarious ones (blue and red respectively). (B) Average number of spikes elicited by single and multiple stimuli for each phase. Gregarious (red): single object 28.64±4.31, multiple objects 48.32±7.61. Solitarious (blue): single object 27.87±5.28, multiple objects 30.31±5.19. ***Planned comparisons revealed a significant difference between the phases in the number of spikes elicited in response to movement of multiple objects and a difference in the response to single versus multiple approaches within the gregarious group (*p*<0.001).
(TIF)

Figure S14 A triple interaction between phase, stimulus type and habituation was not found. Habituation is presented separately for gregarious (dark line) and solitarious (bright line) animals and for each of the different visual stimuli, using regression lines fitted for the log-transformed number of spikes. Under all four visual conditions, the solitarious nymphs’ regression lines were sharper in negative gradient.
(TIF)

File S1 Supplementary Material. Contains the files: Table S1, Table S2.
(RTF)

Movie S1 A short clip showing a 2 minutes example of an experiment in real time.
(AVI)

Movie S2 Stop and go motion in the field. Southern Israel, 2013.
(AVI)

Movie S3 Stop and go motion in the field. Southern Israel, 2013.
(AVI)

Movie S4 A short clip of an experiment with tracking results showing the build-up of motion. x5 speed-up.
(WMV)

Movie S5 A short clip of an experiment with tracking results showing synchronization as a preference to the clock-wise direction. x5 speed-up.
(WMV)

Movie S6 A short clip showing the switching between synchronized states in the detailed model.
(AVI)
