# Supplementary figures and images for: Individual Pause-and-Go Motion Is Instrumental to the Formation and Maintenance of Swarms of Marching Locust Nymphs
Source: PLoS One. 2014 Jul 2;9(7):e101636. doi: 10.1371/journal.pone.0101636 (PMC4079690; doi:10.1371/journal.pone.0101636)

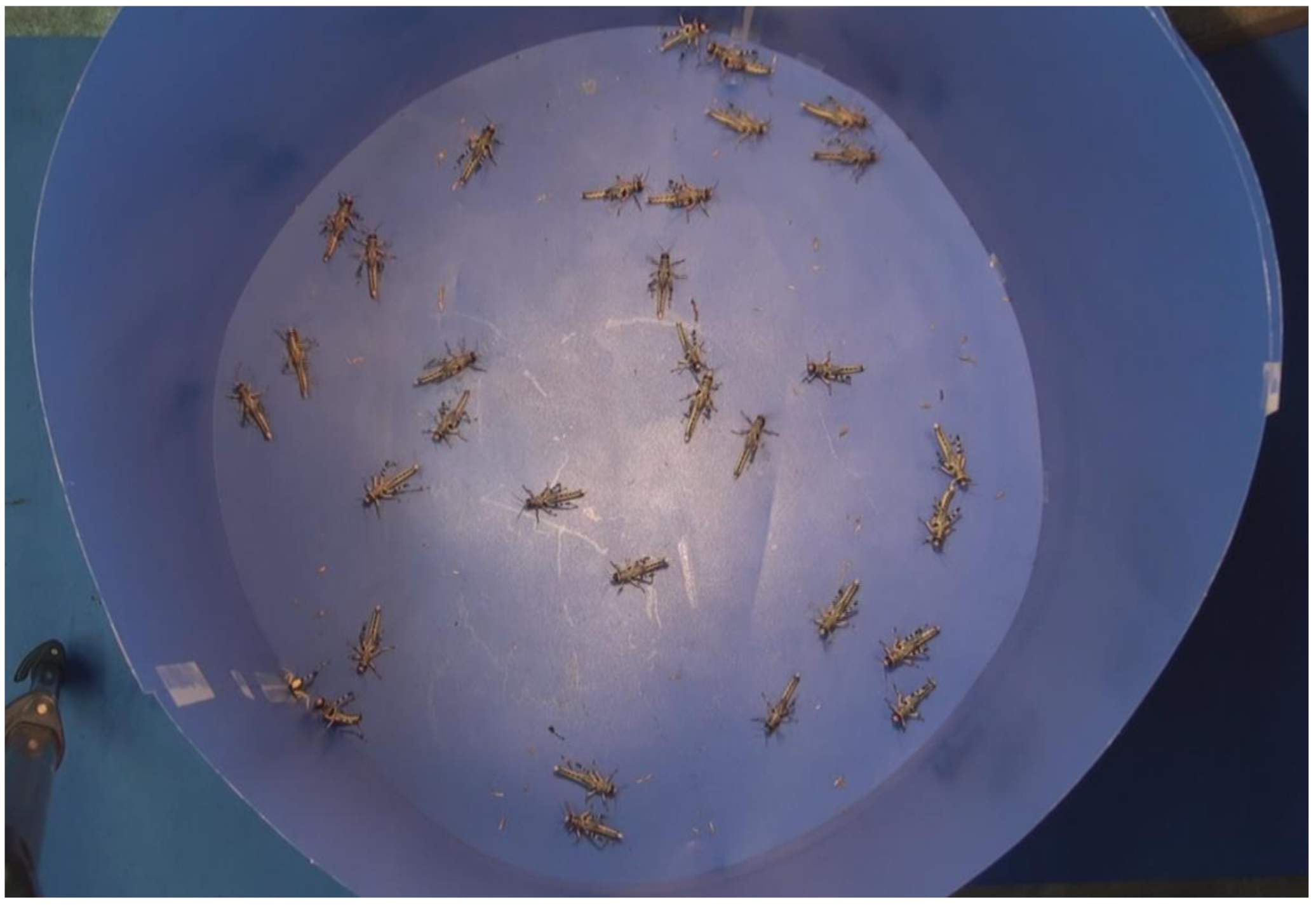

Supplement: Figure S1 — The experimental setup: Several dozen locusts are placed in a plastic circular arena with a diameter of approximately 50 cm. (TIF) [file pone.0101636.s001.tif]

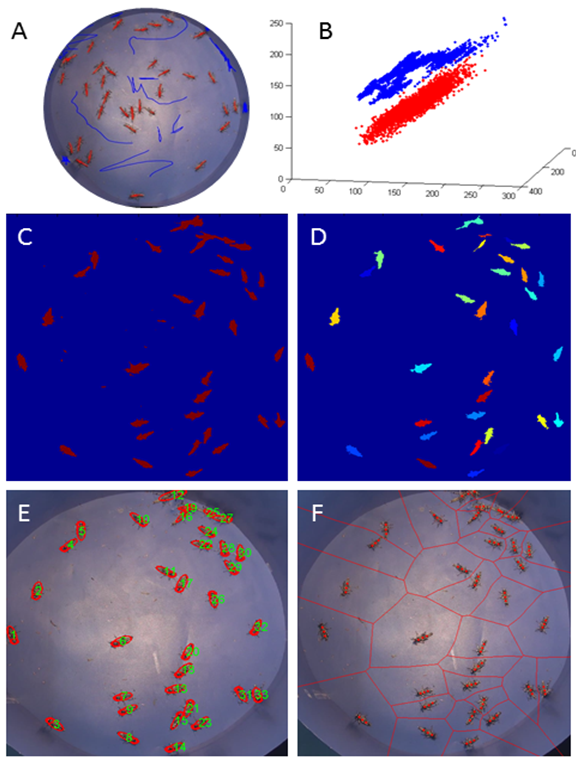

Supplement: Figure S2 — Video analysis algorithm. (A) A hand-labeled frame in which animal were painted red and some of the background was painted blue. (B) The RGB content of labeled pixels. The two clusters are separated using a support vector machine. (C) All pixels in each frame are classified as either animal (red) or background (blue). (D) Labeled regions that fit certain size properties. (E) Numbered objects. (F) Voronoi cells associated with individual animals. (TIF) [file pone.0101636.s002.tif]

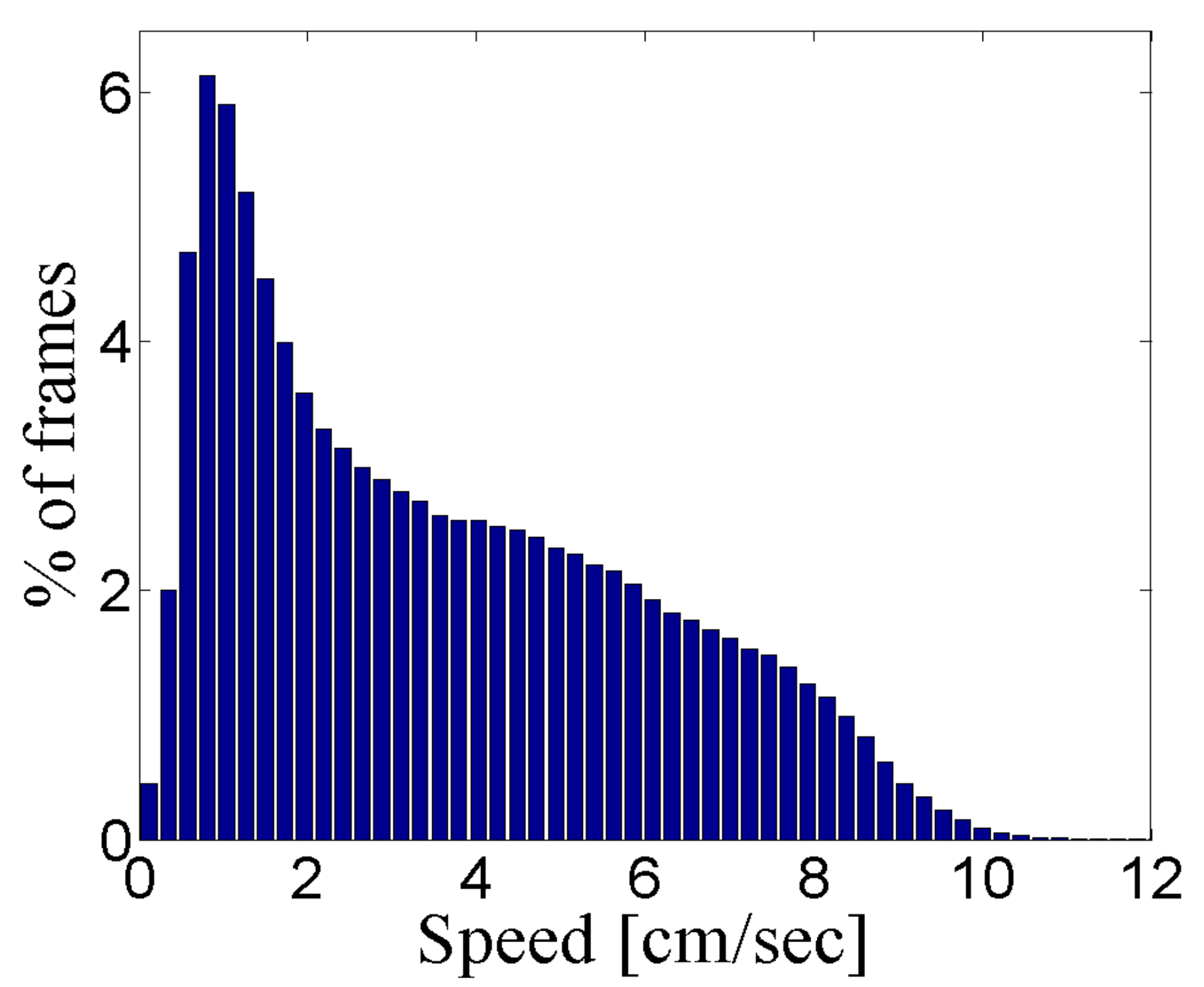

Supplement: Figure S3 — Experimental results: The distribution of walking speeds. (TIF) [file pone.0101636.s003.tif]

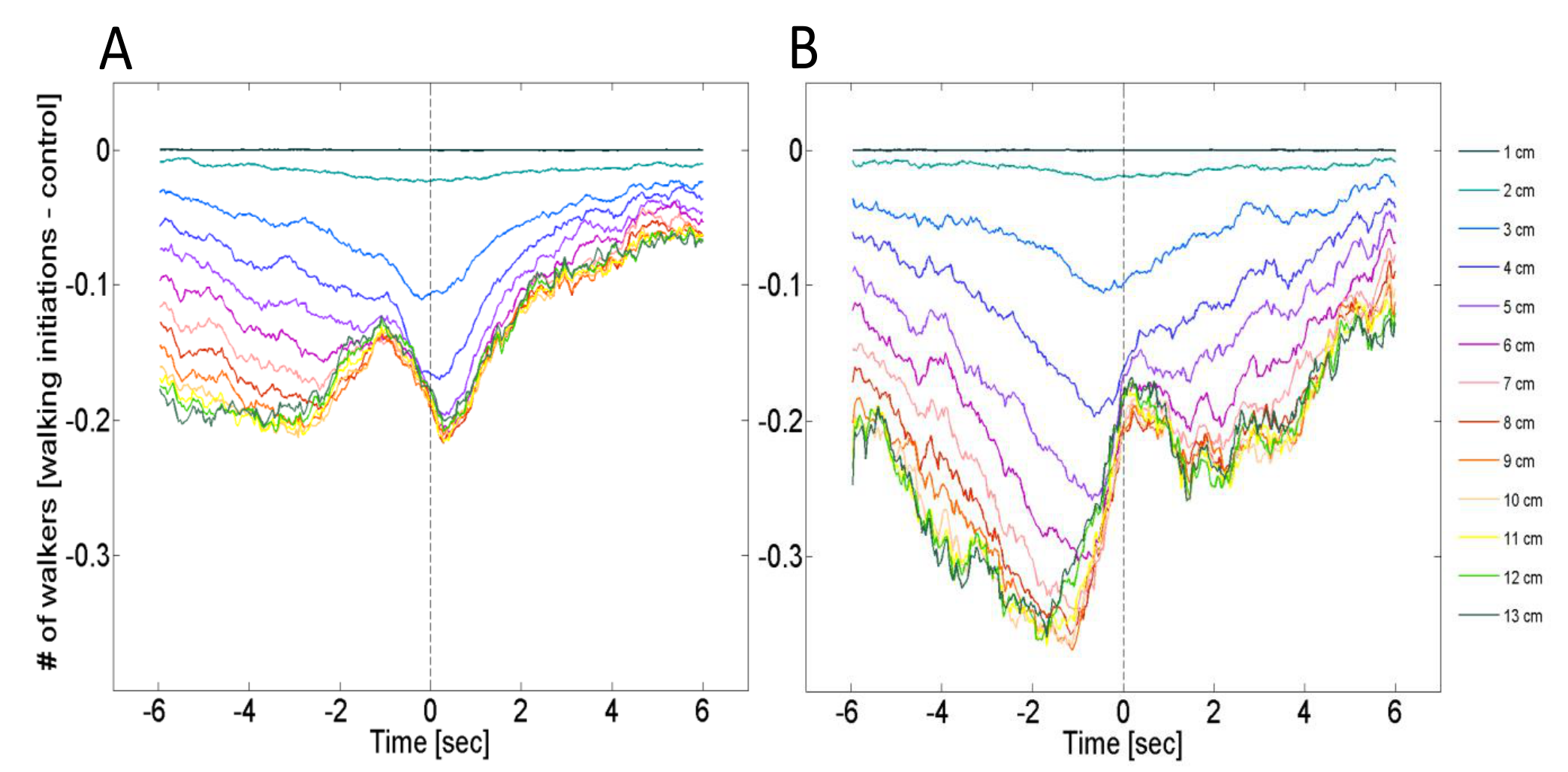

Supplement: Figure S4 — Experimental results. The optical flow in the front (A) and in the back (B) halves of the walking initiating animal’s visual field. Each curve shows the average number of walkers at time t from a walking initiation event within a given distance from the animal that is starting to walk. For example, to generate the purple curve we listed all walking initiation events. Suppose that during event 1, animal k start walking at time . We counted the number of animals walking within a distance of 5 cm from animal k at time and then averaged over all walking initiation events. Other distances we calculated in a similar manner. While in the front, a walking initiation is preceded by a reduction in the number of moving nymphs, an increase in the same parameter is seen in the back. In both halves of the visual field the signal saturates at around 9 cm, suggesting that above this radius no further visual information regarding the animal’s surrounding is obtained. (TIF) [file pone.0101636.s004.tif]

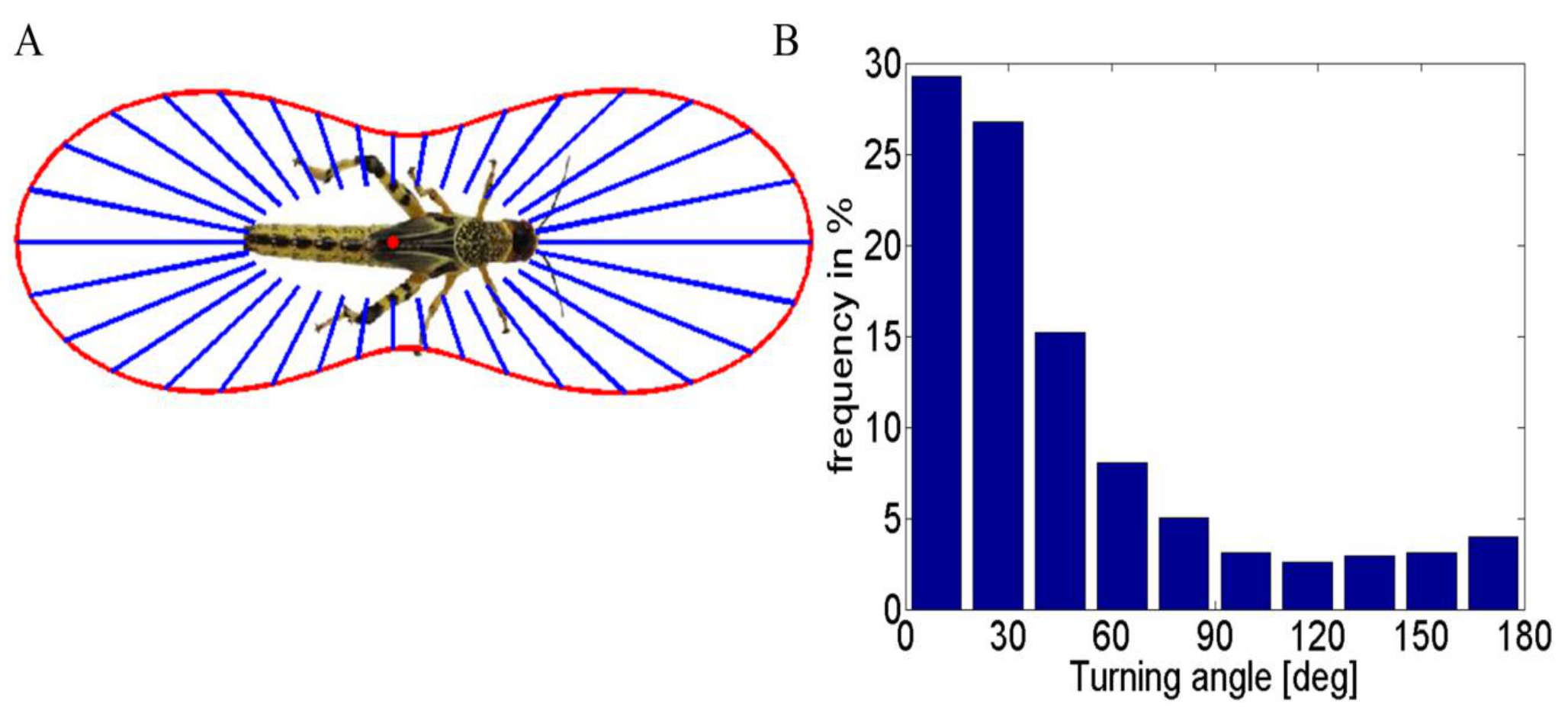

Supplement: Figure S5 — Experimental results: Angles. (A) A histogram showing the distribution of angles in which animals that started walking due to a tactile stimulus were touched. (B) The angle between the head direction 10 frames before walking and the velocity vector five frames after walking started. (TIF) [file pone.0101636.s005.tif]

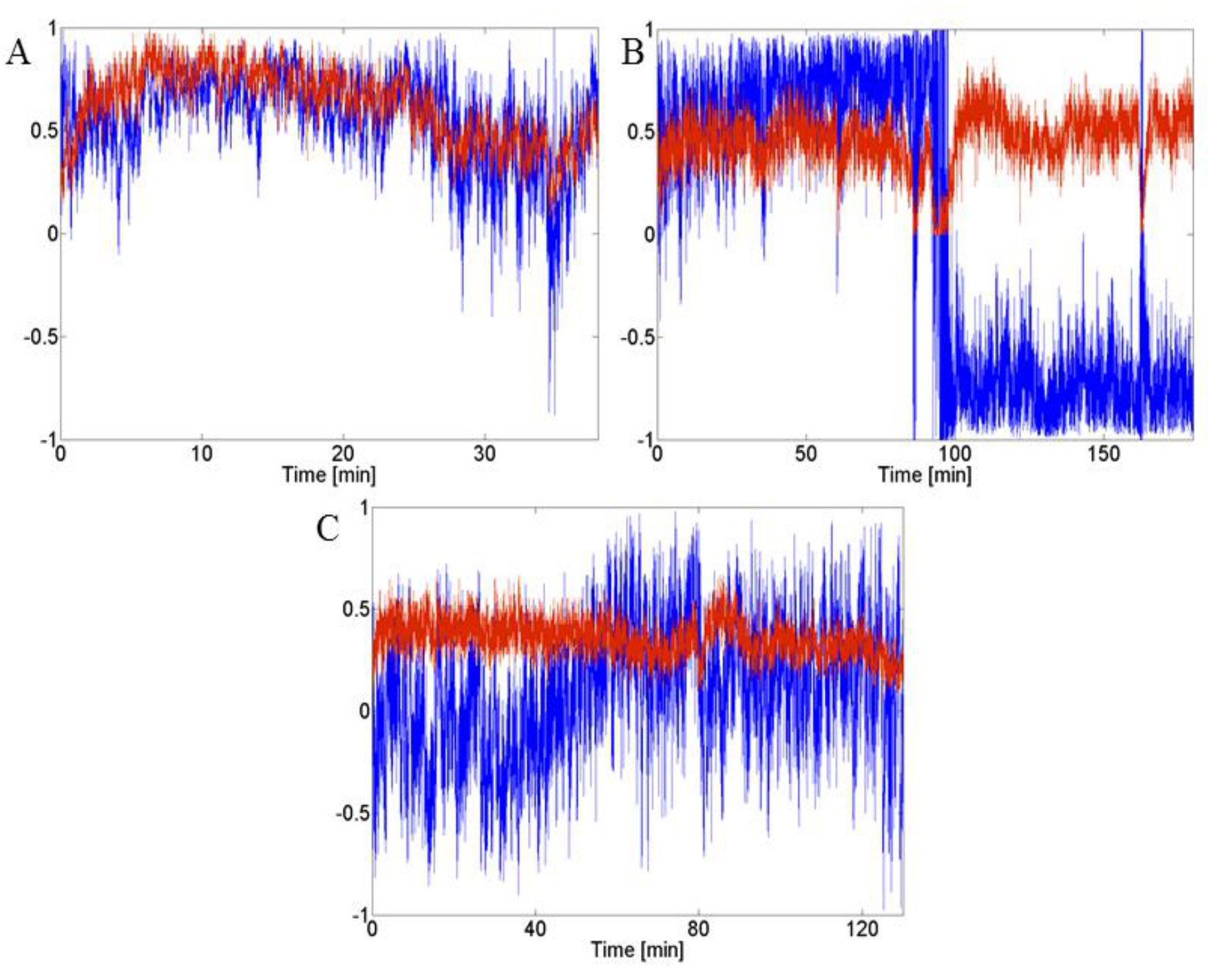

Supplement: Figure S6 — Experimental results. The time-evolution of the order parameter (blue) and fraction of walking animals (red) in the three experiments described in Table 1. The two variables are correlated with an average correlation coefficient of 0.38. (TIF) [file pone.0101636.s006.tif]

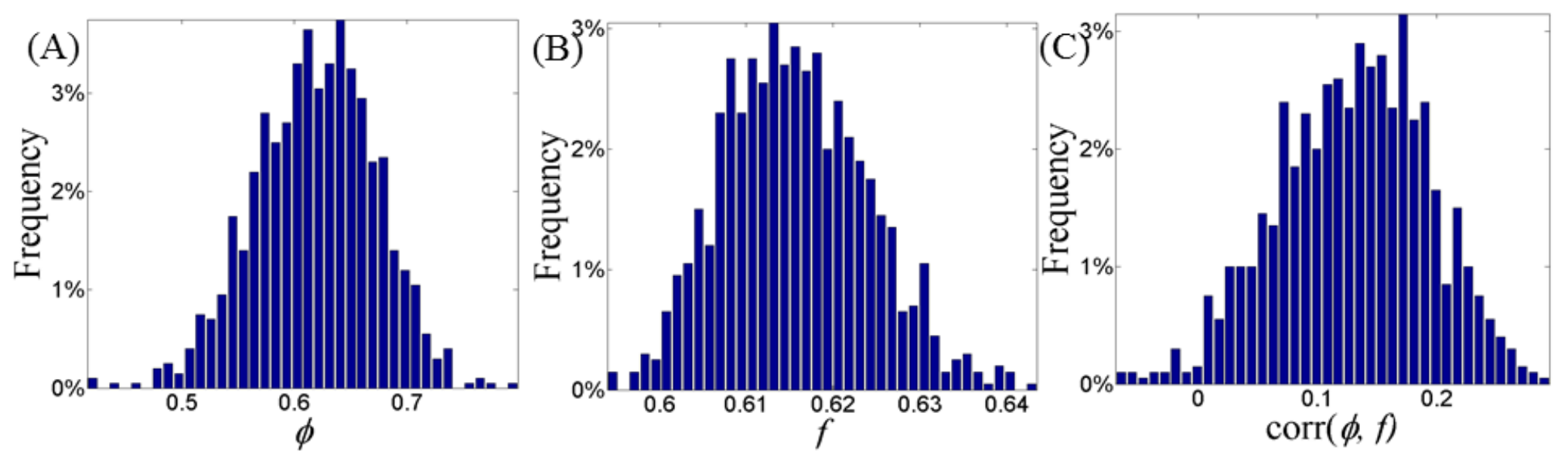

Supplement: Figure S7 — Detailed model results: The distribution of the average order parameter (A), average fraction of moving particles (B), and the correlation between the two (C) in 1500 simulations with length corresponding to 30 minutes of experiment time. (TIF) [file pone.0101636.s007.tif]

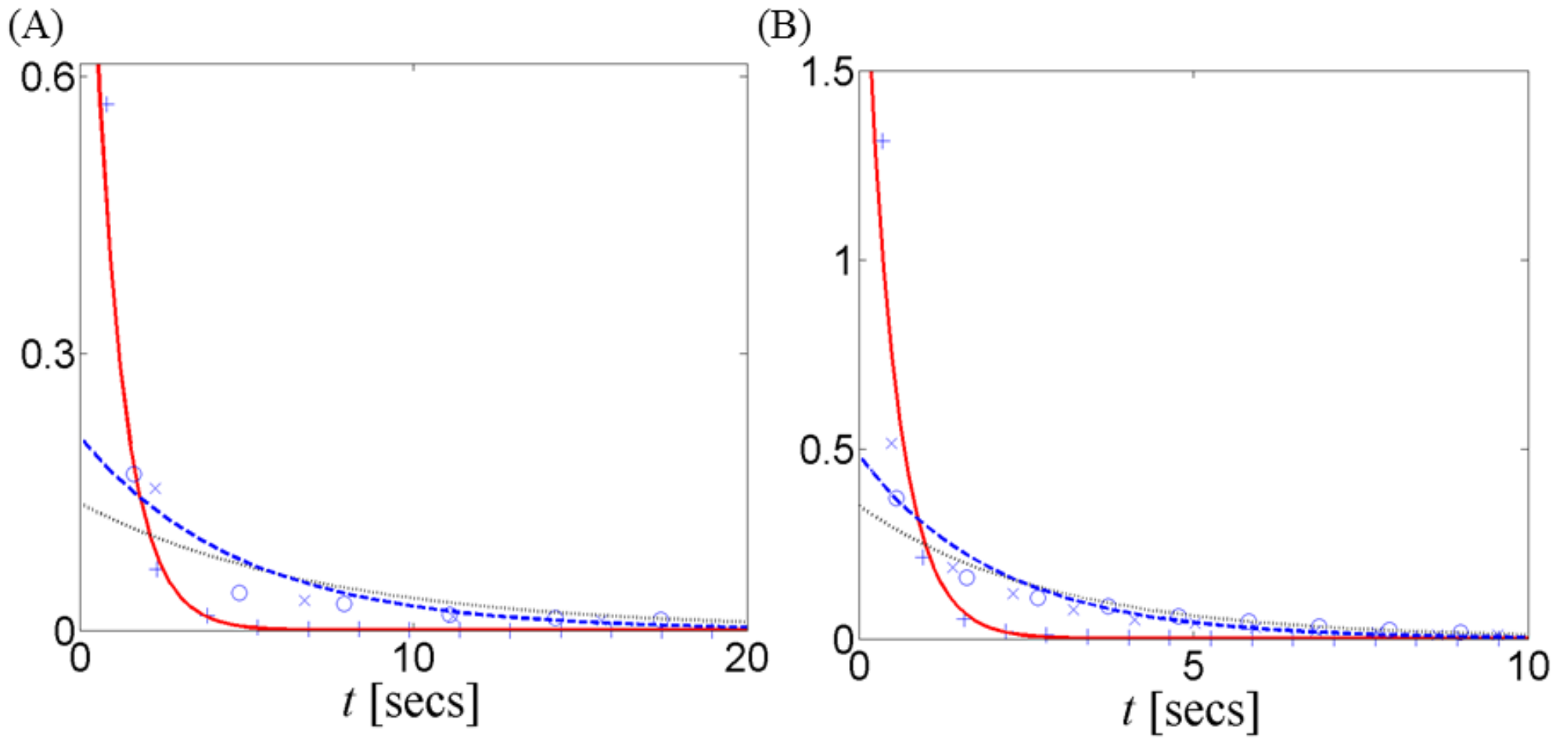

Supplement: Figure S8 — The distribution of waiting times between transitions in the 4-states CTMC approximation. (A) The detailed model. (B) The simplified model.x: state 1, o: states 2 and 4, +: state 3. Solid, dashed and dotted curves are a maximal likelihood fit to an exponentially distributed random variable from states 1, 2+4 and 3, respectively. (TIF) [file pone.0101636.s008.tif]

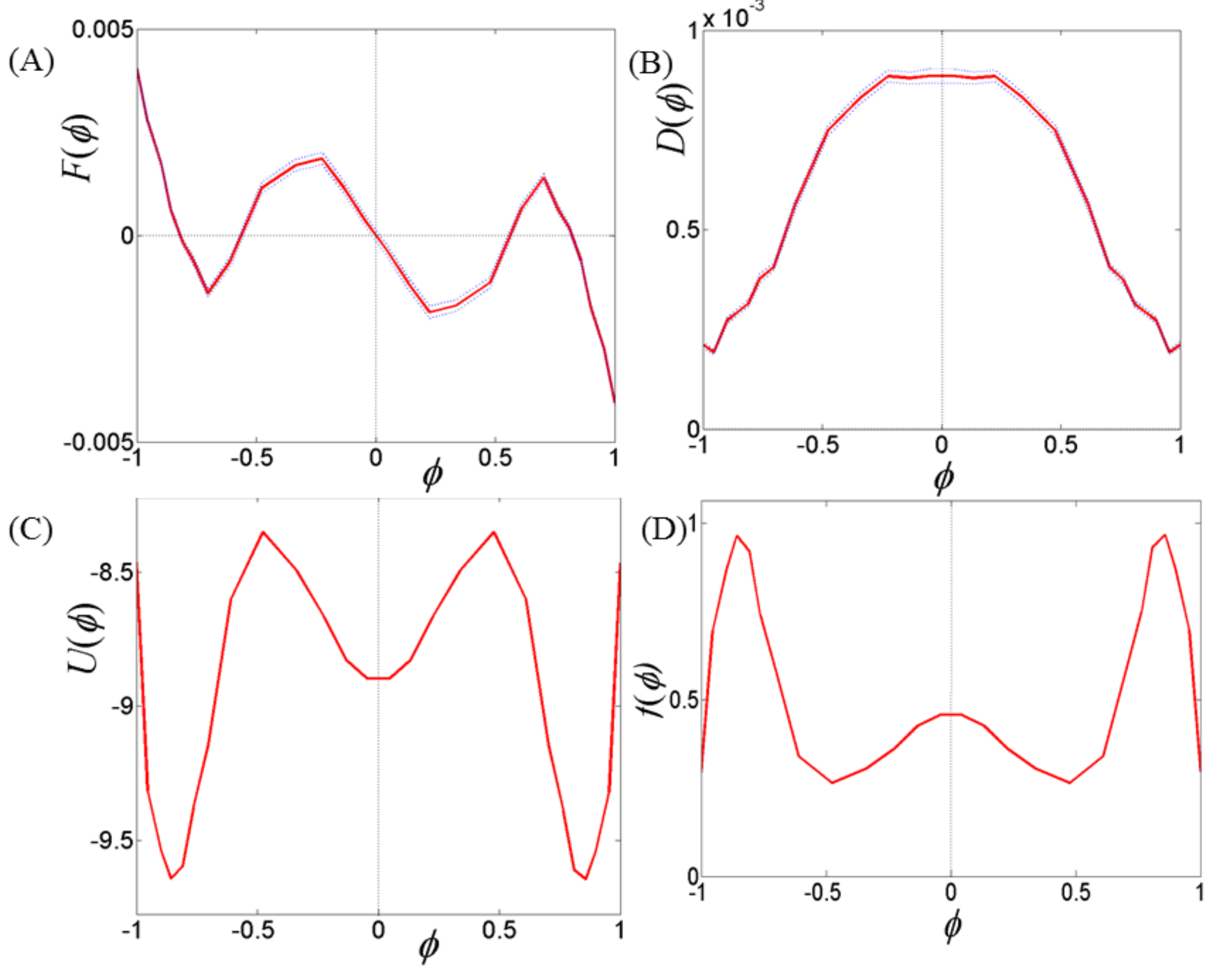

Supplement: Figure S9 — Detailed model results: Parameters for the diffusion equation describing the effective dynamics of the order parameter , showing three meta-stable states. (A) The drift, Fφ with three stable roots. (B) The diffusion coefficient, Dφ has a maximum around zero. (C) The potential, Uφ, and (D) The probability density function, fφ. (TIF) [file pone.0101636.s009.tif]

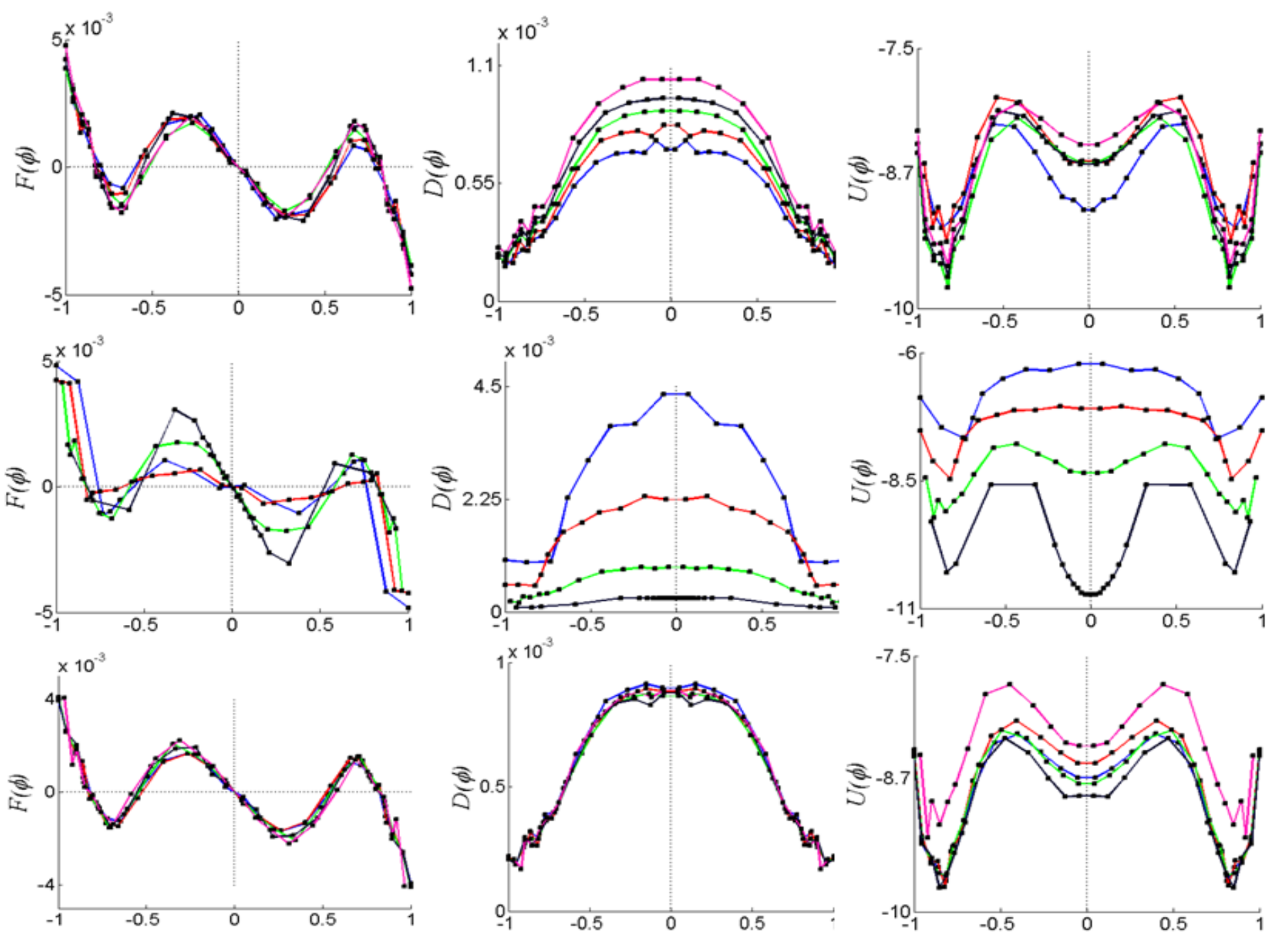

Supplement: Figure S10 — Variation of parameters in the detailed model. Top row: number of particles 10 (blue), 15 (red), 34 (green-experimental value), 100 (black). Middle row: Probability to V = 0.1 (blue), 0.2 (red), 0.3674 (green-experimental value), 0.4 (black), 0.5 (purple). Bottom T = 0.02 (blue), 0.03 (red), 0.039 (green-experimental value), 0.05 (black), 0.06 (purple). (TIF) [file pone.0101636.s010.tif]

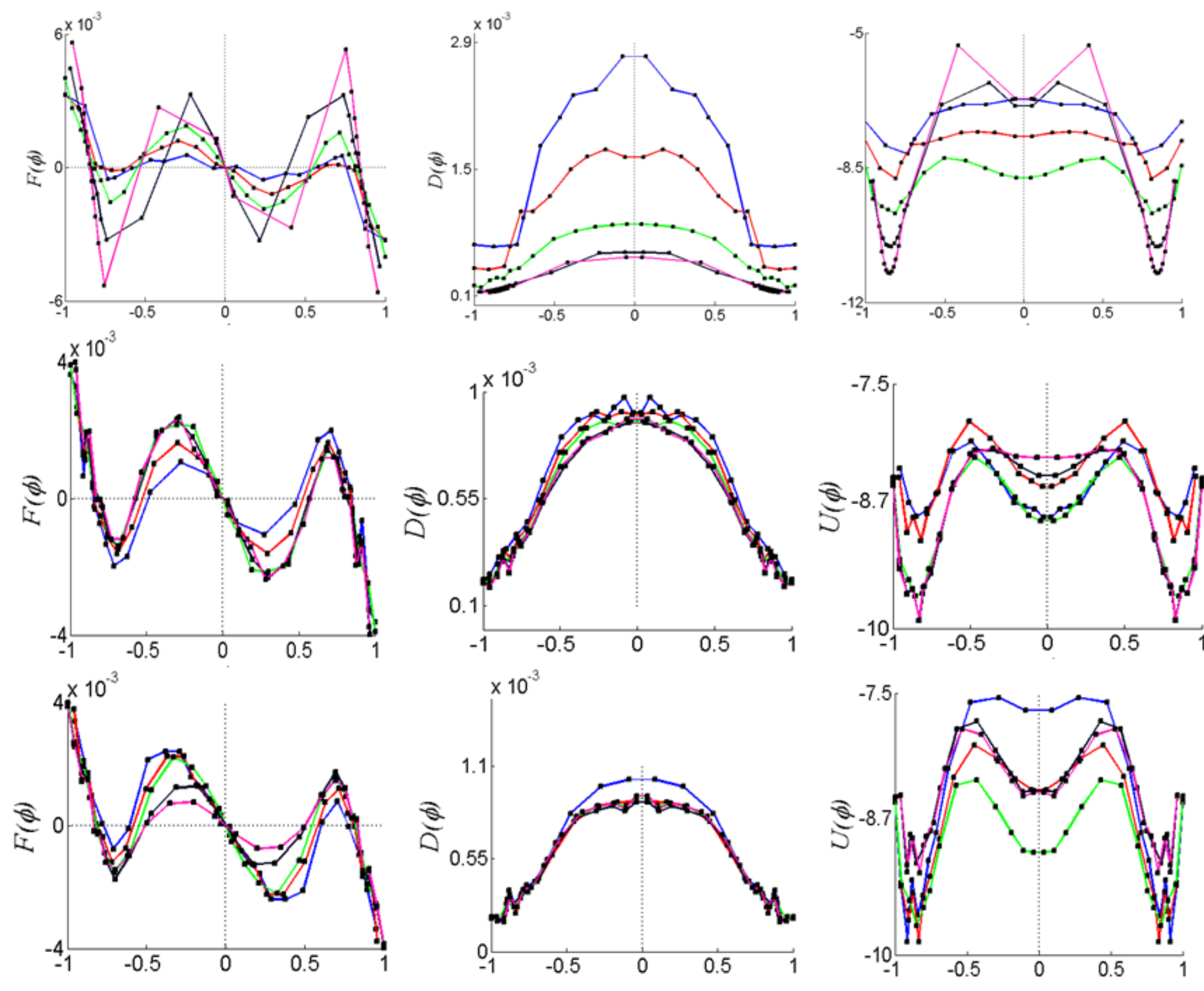

Supplement: Figure S11 — Variation of parameters in the detailed model. Top row: Probability to stop moving = 0.3 (blue), 0.4 (red), 0.5083 (green-experimental value), 0.6 (black), 0.7 (purple). Middle row: . 10 (blue), 15 (red), 34 (green-experimental value), 50 (black),70 (purple). Bottom row: Interaction radius r = 3 (blue), 4 (red), 5 (green-experimental value), 6 (black), 7 (purple). As changes the average number of interacting neighbors, this is also equivalent to changing the interaction thresholds and . (TIF) [file pone.0101636.s011.tif]

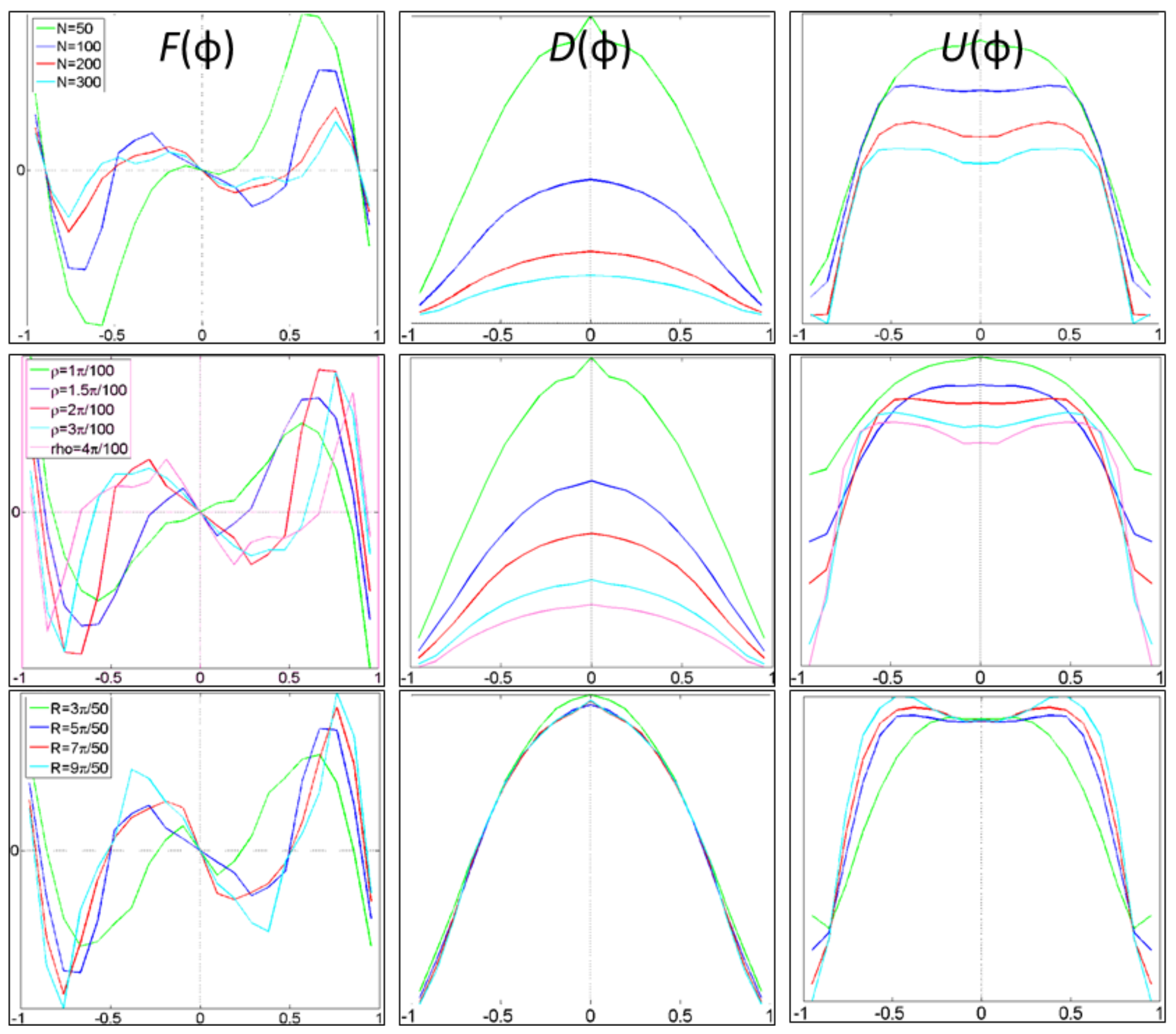

Supplement: Figure S12 — Variation of parameters in the simplified model. Left column: effective drift, middle column: effective diffusion and right columns: effective potential. Top row: dependence on , Middle row: dependence on and bottom row: dependence on . (TIF) [file pone.0101636.s012.tif]

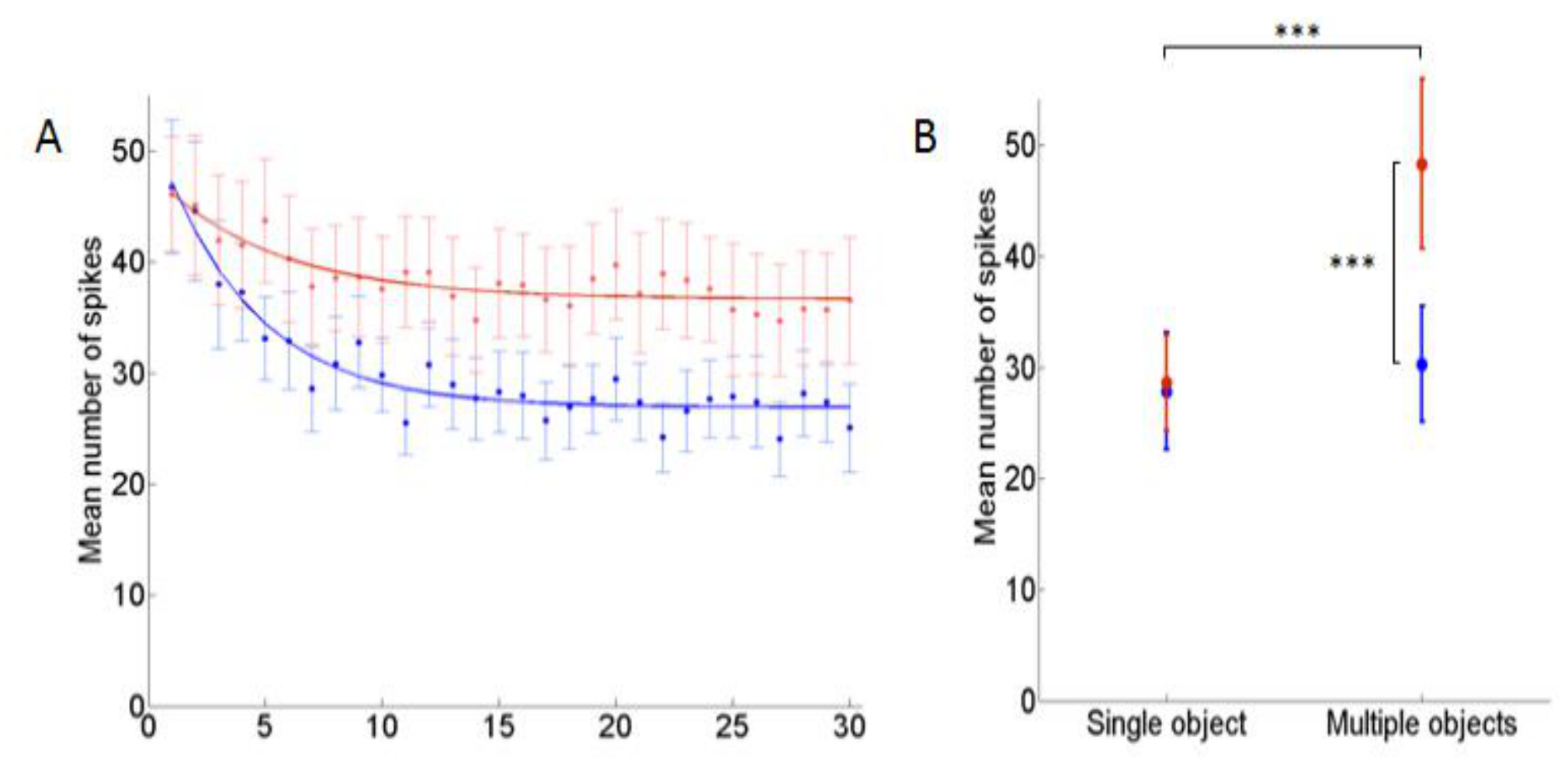

Supplement: Figure S13 — Phase related differences in DCMD response. (A) The habituation observed in the elicited number of spikes was more substantial in solitarious nymphs than in gregarious ones (blue and red respectively). (B) Average number of spikes elicited by single and multiple stimuli for each phase. Gregarious (red): single object 28.64±4.31, multiple objects 48.32±7.61. Solitarious (blue): single object 27.87±5.28, multiple objects 30.31±5.19. ***Planned comparisons revealed a significant difference between the phases in the number of spikes elicited in response to movement of multiple objects and a difference in the response to single versus multiple approaches within the gregarious group (p<0.001). (TIF) [file pone.0101636.s013.tif]

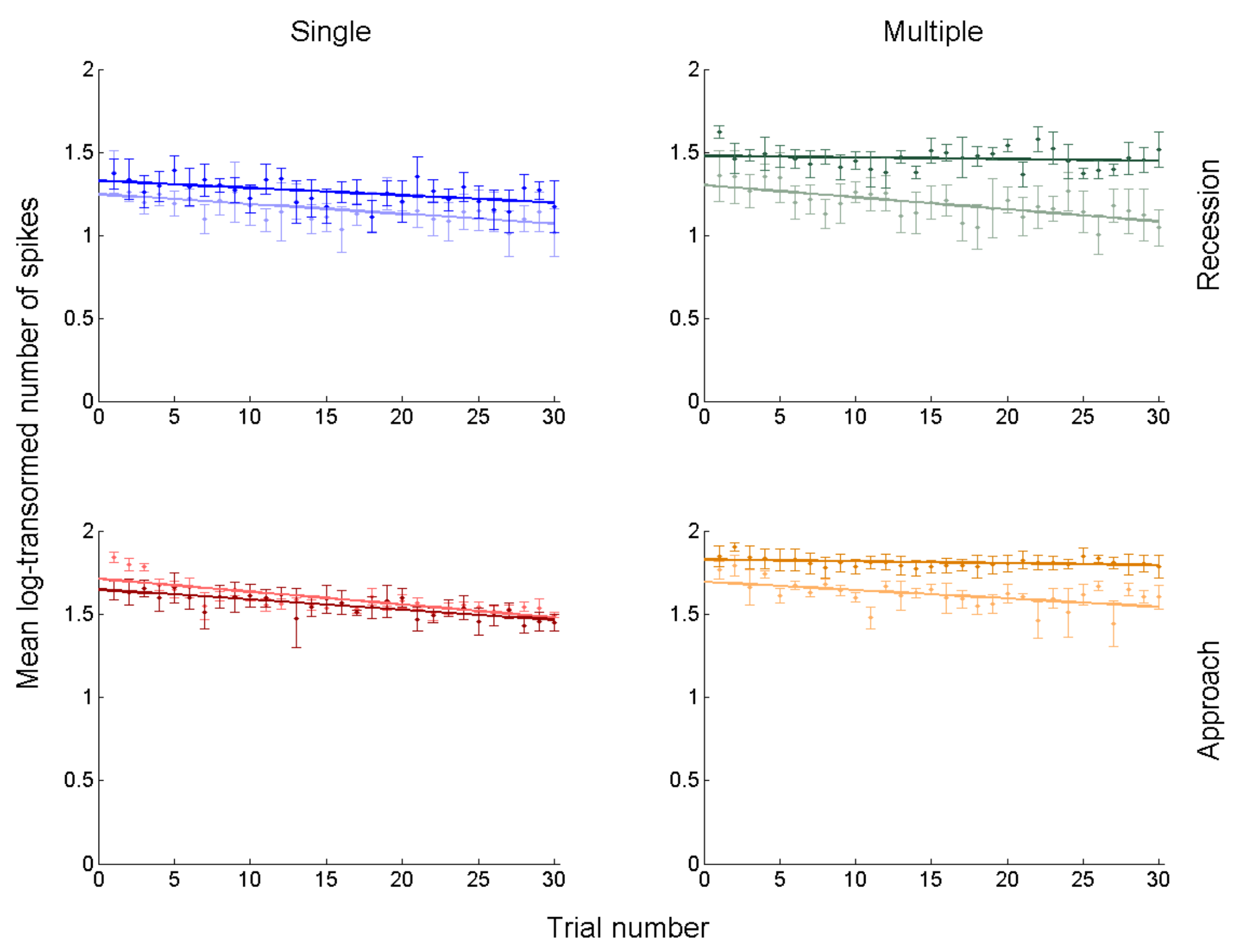

Supplement: Figure S14 — A triple interaction between phase, stimulus type and habituation was not found. Habituation is presented separately for gregarious (dark line) and solitarious (bright line) animals and for each of the different visual stimuli, using regression lines fitted for the log-transformed number of spikes. Under all four visual conditions, the solitarious nymphs’ regression lines were sharper in negative gradient. (TIF) [file pone.0101636.s014.tif]
