# Supplementary material for: Individual Pause-and-Go Motion Is Instrumental to the Formation and Maintenance of Swarms of Marching Locust Nymphs
Source: PLoS One. 2014 Jul 2;9(7):e101636. doi: 10.1371/journal.pone.0101636 (PMC4079690; doi:10.1371/journal.pone.0101636)
Supplement: File S1 — Supplementary Material. Contains the files: Table S1, Table S2. (RTF) [file pone.0101636.s015.rtf]

Supplementary Information

Multi-target tracking

The video processing algorithm is divided into three stages: pre-processing, object identification and post-processing. The algorithm was implemented in Matlab.
1.	Pre-processing: Movies are converted into a sequence of jpeg images using the software virtualdub.
2.	Object identification: Each frame in the movie is analyzed to find the number of animals and their instantaneous positions and orientations. See below for details.
3.	Post-processing: This stage consists of four parts.
i.	Identifying which individual in one frame corresponds to which in subsequent frames. 
ii.	Identifying periods of motion and rest using a repeated moving median method.
iii.	Smoothing trajectories and head orientations using a LOWESS method (Hen et al., 2004).
iv.	Calculating velocities.

Object identification: Video and image analysis of the sequence of images making up a movie is performed in three layers related to pixels, objects and post-processing.

Pixel analysis: Every pixel in every frame has an RGB color content which consists of three numbers from 0 to 255, showing the intensity of Red, Green and Blue in the pixel. Since gregarious locusts are typically yellow, grey and black, while the background of the arena was blue, the color content of each pixel can be used to classify pixels into “animal” or “background”. To this end, a single frame is taken and analyzed by hand. Specifically, a few animals are painted red while some area of the background is painted blue. FigureS2A shows one such painted frame and Figure S2B for the RGB content of the pixels, before it was painted.
One can see that the RGB content of the pixels consists of two clusters, as depicted in figure S2B. The classification problem is solved using a Support Vector Machine with a quadratic kernel applying the training and classification functions in Matlab's bioinformatics toolbox.  Using this technique, each frame can be represented as a binary matrix showing which pixels are suspected to be a part of an animal and which are background. See figure S2C for an example.

Objects analysis: The result of the previous layer is a binary matrix for each frame.  This matrix is noisy and the classification of pixels is often incorrect. To this end animal pixels are united into connected regions using the regionprops function in Matlab's image processing toolbox. Each region is smoothed and analyzed according to size and area. Objects with shapes that are uncharacteristic of locusts are discarded. Figure S2D shows a labeled image and the remaining objects.

Post-processing: Objects are identified and numbered relative to locations on the previous frame (figure S2E). Then, we form a list of points in the arena, in which three points are placed along the principle axis of each animal.  Figure S2F shows the Voronoi graph of such points where all edges passing inside animals are omitted. The resulting graph is a good characterization of the space each animal occupies. The edges of the graph represent a boundary between animals and are used to separate animals in the subsequent frame. This is a new feature that increases the success of the tracking algorithm to continuously follow individuals even at very small separations.


Detailed model and simulations
Previous experiments show that bands of marching locusts can spontaneously switch between states of coherent motion (Buhl et al., 2006). This occurs even without any apparent reason, implying it is an intrinsic property of the dynamics. Similar dynamical transitions can be observed in other organisms (Tunstrom et al., 2013) and artificial models (Bode et al., 2010; Topaz et al., 2012). Thus, understanding the reasons for these sudden transitions in coherent motion is crucial for understanding the dynamics and the emergence of order and disorder in general, and in locust movement in particular. We propose a one dimensional model which captures the essential dynamics and interactions obtained by our detailed analysis of experiments. This detailed model successfully reproduces movement patterns that quantitatively agree with experimental ones. The model offers increased flexibility in analyzing different situations and dynamical regimes, which are inaccessible by experiments, in particular due to limited sampling. 

The model consists of N particles representing individual locusts that move in a one dimension circular domain with radius R, modeling an annulus in which the locusts effectively move. Both the time and position of particles are discretized, where each time step corresponds to a single frame in the recorded experiments () and the circular domain is discretized uniformly with grid points  such that the grid spacing  is the average walking distance between frames. See Figure S3 for the distribution of walking speeds. The state of the system is defined by the positions, , head orientations  corresponding to clockwise (CW) or counter clockwise (CCW) direction, and a Boolean variable indicating whether particles are moving or not, , respectively. Hence, in our model all particles either stand or move at a constant speed –at each time step moving particles shift one grid point either CW or CCW depending on the head orientation. In this respect, the model is similar in spirit to traffic cell models. The model is also related to the a-synchronous model suggested by Bode et al. (2010). The dynamics is determined by specifying the probability of a particle to start or stop moving and to change its head direction (turn). Figure 3B shows a snapshot from simulation.

Initial conditions: Each simulation was initialized with a uniform distribution on the entire states space.

Stopping: Following experimental observations, a moving particle can stop due to two reasons: spontaneously, with probability , or by colliding with a different particle, i.e. tactile stimulus, with probability . Assuming independence, the overall probability for particle  to stop is 
,
wheredenotes the characteristic function of an event , i.e.,
.

Walking initiation: Following experimental observations, standing particles can start moving due to three reasons:
·	Tactile stimulation: After being touched by other moving particles. Movement will occur with a probability .
·	Visual stimulation: when particles in front of the locust move away or when particles behind it move closer. Movement will occur with a probability  .
·	Spontaneously: without any apparent stimulation, with probability  .
Assuming independence, the overall probability for particle  to stop is 
,
Here, is the sum of speeds of particles behind the animal (relative to its orientation) up to an interaction distance, is the sum of speeds of particles in front of the animal up to an interaction distance ,  and  are threshold constants. More precisely,
.
The dependence on and  is suppressed for short hand. In words, is the set of particles moving within a distance  from particle, and are partitions to the back and front of particle, respectively.

Turning: As observed in experiments, particles can change their direction only by stopping and then turning. This gives a more detailed representation of locust movement than other models that assume continuous movement and turning. A particle which is starting to walk has a given probability of turning. This probability depends on its orientation relative to the order parameter. We recall that the order parameter is defined as

whereis the set of moving particles at time ,
,
and denotes the number of elements in a set , i.e.,  is the number of moving particles at time t. Note that the fraction of walking animals is given by
.
As observed in the experiments, the probability of turning depends on whether a particle is oriented with or against the crowd. For simplicity, we assume a linear dependence on

Note that as there is no preferred direction and the probability is regardless of orientation.


Simulation results: 
Simulation parameters were scaled to fit experimental values and All parameters values were fitted according to statistics obtained from the experimental data: ,,. Figure 3C shows the time evolution of the fraction of walkers and the order parameter in one typical simulation.

Simulations and comparison with experiments enables seeing whether the model provides a good description of the actual behavior. All averages are calculated from 1,500 simulations, each simulation corresponds to an experiment of about 30 minutes long.

Order parameter: In its active state (high) the system has three meta-stable states; two ordered states in which   is close to 1 and a disordered state around . The average order parameter (in absolute value) is 0.62 with a standard deviation of 0.05, which is consistent with the experimental value of 0.56 (See figure S10A).

Number of walkers: The average fraction of moving particles is 0.62 with a standard deviation of 0.01, which is consistent with the experimental value of 0.62 (figure S10B).

Correlation between the order parameter and the fraction of walkers: As in the experiment, the order parameter is positively correlated with the fraction of moving particles; the average correlation is 0.13 with a standard deviation of 0.06. The experimental value (0.38) is slightly higher (figure S10C).

Coarse-graining 
As discussed in the main text, one of the fundamental observations in this paper is that the dynamics can be divided into four meta-stable states, depicted in Figure 3A: A relatively static state, in which most of the animals are standing, and three active states, in which most of the animals are walking. The three active states can be classified according to the order parameter and correspond to one disordered and two ordered movement patterns - CW and CCW. In order to verify this assumption we divide the states space into four well-separated regions according to , the fraction of walking particles, and , the order parameter:
1.	Low movement: .
2.	High movement, ordered CCW rotation:  and .
3.	High movement, disordered state:  and .
4.	High movement, ordered CW rotation:  and .
The fraction of time that the system spends in each of the states along with the transition probabilities are given in Figure 3A.

One of the tests for verifying our assumption that the complex dynamics of the system can indeed be approximated by a continuous-time Markov chain (CTMC) is that waiting times between transitions should be independent and exponentially distributed. Figure S8A shows the density function of waiting times along with the maximum likelihood fit to an exponential variable.

A continuous approximation for 
In order to further verify which meta-stable states the system has, the effective dynamics of  is approximated by a diffusion process of the form
,
Where is a Weiner process and  and are the drift and diffusion, respectively. Following Yates et al. (2009), and can be approximated from the dynamics as
,
Wheredenotes averaging over all frames in which the order parameter is in some neighborhood of.
The invariant density of the process can be written as (Gardiner, 2004)
,
where the potential, is given by

Figures 4B and S11 show the parameters of the effective diffusion as obtained from simulations. Compare also with the experimental result, Figure 4A. The potential has three local minima, two at high values of  (), corresponding to the ordered states, and a shallower one at. This implies that on average, ordered states persist for long periods. This can also be observed from the shape of  depicted in figure S11D. The fourth meta-stable state, relating to low values of, cannot be observed since this method averages over all values of. This implies that the effective diffusion equation for  does not reveal the full coarse-grained behavior of the system.

Different parameter values
In order to test the model's sensitivity to parameters as well as study swarming in conditions other than those in our experiments, we vary some of the model parameters. In particular, we are interested in understanding which parameters may "ruin" one of the metastable states –either the ordered or disordered ones. We find that all states are robust and do not depend on fine tuning of parameters. However, the probability to find the system in a disordered or ordered state as well as the transition rates between them may vary. 

A few interesting observations are worth noting.
-	If the system is too dilute, the disordered state may not be stable (top row of Figure S12). This can be explained by noting the particularly high diffusivity at small values of  in this case.
-	If the probability to start moving following one of the stimuli is high than disordered state may not be stable (middle and bottom rows of Figure S12). This may be due to the fact that any small fluctuation immediately brings almost full synchronization.
-	If the system is too large the ordered state may not be stable (middle row of Figure S13). This unintuitive result can be explained by the fact that with a large system, it is difficult to achieve full synchronization with a local interaction. As a result, the probability to turn towards or against the swarm remains fairly similar. This behavior is an artifact of the global definition of  used in the detailed model and does not exist in the simplified, local model presented below.


Simplified, macroscopic model and simulations

In order to identify the key principles leading to the kinetic order-disorder transition as predicted by our experiments with marching locust bands, the detailed model described above was simplified, stripping it from many of the experimental details. In addition, all interactions between particles were taken to be local. This implies that a particle does not depend on the order parameter of the entire system, but only on a local version that considers a few close neighbors.  
Similar to the previous section, the model consists of N particles representing individual locusts that move in a one dimension circular domain. However, since we are more interested in the characteristics of the dynamics at a large number of particles, the domain of simulation is taken to be the segment  with periodic boundary conditions.Except for this difference, the state of the system is defined by ,  and ,  and , as described in the previous section. We recall that, denotes the set of moving particles within the interaction distance, 
.
Similarly,  denotes the number of neighbors of, i.e., the number of elements in .
The dynamics of the system is again specified by the probabilities of particles to start walking, stop or turn. 
·	Stopping: Spontaneous only with probability.
·	Starting: Either spontaneous or locally triggered. The key characteristic of both the tactile and visual walking stimulus described in the experiments and the detailed model is that the frequency an animal is exposed to the stimulus increases with the number of animals walking in its vicinity. Thus, we define an increased probability for starting to walk whenever the number of moving particles is higher than a given threshold. Assuming independence, the overall probability a standing particle will start moving is
.
·	Turning: Particles turn only when switching from a standing () to a moving state (). The probability of turning depends linearly on the local order parameter(with a minimum)
,
where
.


Simulation and results
We report results of simulations preformed with  particles and an interaction radius. Since the length of the arena is set to, the interaction length, which is 10 times smaller, has on average 5 particles. We stress the fact that in this simulation, all interactions and events are local. In other words each particle senses only a range. Other parameters are , , ,, and . 
It is well know that locusts, which were reared at different conditions, respond differently to their conspecifics. In order to test the impact of different animal-animal interactions on the dynamics of the marching band, we vary the slopes of the turning probabilities and . In particular, we demonstrate that three dynamical regimes are possible for : a single disordered state, two ordered states, or all three (1 disordered + 2 ordered). In addition, the low mobility (small ) is always meta stable. Figure 4C depicts the effective drift in these three regimes. The effective diffusion is similar to results obtained with the detailed model (see Figure 4B). Parameters areand
1.	Single disordered state: .
2.	One disordered and two ordered states: .
3.	Two ordered states: .

Focusing on the latter case, in which both ordered and disordered states coexist, we demonstrate again that the dynamics can be approximated by continuous-time Markov chains with four states:
1.	Low movement: .
2.	High movement, ordered CCW rotation: and .
3.	High movement, disordered state: and .
4.	High movement, ordered CW rotation: and .

Tables S2 shows occupancy frequencies and transition probabilities. Values are rounded to the nearest 5%. Figure S7B shows waiting times between transitions and a maximum likelihood fit to exponential random variables. We find that the fit to a CTMC is improved. Interestingly, the time the system spends in the small regime decreases, but its effect on the dynamics increases as most of the transitions between meta-stable states happen through it. Simulations with larger yield similar results. In fact, the four-state CTMC approximation becomes more accurate.

Different parameter values
Finally, in order to test the model's sensitivity to parameters, we test the effective slow dynamics as a function of the number of particles, , the density, , and the interaction radius, . Figure S14 shows that as the system becomes denser or larger, the dis-ordered state becomes more pronounced. However, the dependence of the interaction distance  is small as long as it is above some critical value. 


Electrophysiology

Dissection and recording: Experiments were performed on 8 fifth-instar locust nymphs (S. gregaria), four approaching the solitarious phase, and four approaching the gregarious phase. Within each phase-group, half the animals were males and half females. Locusts were anaesthetized in CO2 and all six legs were amputated. Each locust was then placed upside down over plasticine shaped to suit its body size, and was secured using pins located along the sides of the body. The head was gently pulled forwards and stabilized using pins. The soft cuticle of the neck connectives was dissected away to reveal the underlying cervical connectives, each of which include a DCMD axon. Fat tissue and tracheas were cleared and locust saline was added as necessary to keep the tissue submerged. The locusts were turned upside up following electrode placement and was left for 5 min before any records of responses to visual stimuli were made.
Extracellular recordings of the DCMD spikes were made with silver hook electrodes (50 mm diameter, A-M systems, Carlsborg, Washington, USA) placed under the left connective and insulated using Parafilm (Pechiney Plastic Packaging Company, Chicago, Illinois, USA) and petroleum jelly. The spikes have the largest amplitude in the connective and have a characteristic response to visual stimuli. Experiments were carried out at 24–28°C. Signals were recorded at a sampling rate of 20 kHz using a differential amplifier (Model 1700, A-M Systems) and were stored on the computer using an A-D board (Digidata 1200, Axon Instruments, Inverurie, Scotland) and Axoscope software (Axon Instruments). The recorded data were analyzed using DataView (W. J. Heitler, University of St. Andrew, http://www.st-andrews.ac.uk/~wjh/dataview/).
Visual stimulation: Animals were mounted in between two 18.5″ LED monitors (Samsung SyncMaster S19B300, Seoul, South Korea) with a display area of 230 mm (vertical) x 410 mm (horizontal), which were positioned perpendicularly to the locust's long axis. The right eye was aligned with the center of both screens in azimuth, positioned 100 mm away from both screens and 10 mm above the bottom of the screens' surface, subtending 128×71 deg (Figure S8A). Each monitor had a resolution of 1366 x 768 pixels, a spatial resolution of 0.3 mm and a refresh rate of 75 Hz. The luminance of the monitors was 250 cd/m2.

Both monitors were controlled by a single personal computer. Stimulation programs were written in MatLab. Stimuli were based on visual interactions observed in the behavioral experiments and were composed of dark squares moving against a bright background with constant velocity of either 2or 8.4cm/sec (representing two extremes of the speed range of marching nymphs in our arena), either receding or approaching the experimental animal. The dimensions of the images ranged from a minimum of 8 (width) x 4 (height) mm to 85 x 15 mm. These proportions correspond to visual angles of 4.58° to 47.7° in width and 2.29° to 8.58° in height. 

Based on Gabbiani et al (1999), the rate of expansion of the object on the eye is determined by the ratio between the half size of the object (l) and the approach velocity (v), i.e., l/. For a constant approach velocity, the angular extent increases slowly at first and then more rapidly as the object nears the eye. We simulated objects with a transversal value of l/= 506 or 2125 msec and a height value of l/= 89or 375 msec, for the fast and slow objects respectively. These values, describing very small and slow objects, are significantly higher than those usually used for looming objects.
Four different visual stimuli were presented to each nymph. Each animal was exposed to 30 repeats of each visual stimulus, giving 120 stimuli in total. One recording of a solitarious nymph's DCMD response to a single receding object was omitted due to low signal to noise ratio. A square started its simulated movement in a 10 seconds long stationary position. It then approached or receded for 6 seconds and stopped again, remaining stationary for 10 seconds. Five seconds separated one stimulus from the next. All objects reached both the minimal and maximal size described above.
Data analysis: Spike times were obtained by applying a threshold to the recorded potential traces to detect the largest spike, which was always that of DCMD. Spike times are defined and expressed here as "time relative to maximal object size", which is defined as time 0. These data were used to calculate the number of spikes and peak firing rate elicited by each visual stimulus and to construct raster plots and peristimulus time histograms. To examine the time course of the response in DCMD, the spike times were transformed to instantaneous spike frequencies by applying a 20 ms Gaussian smoothing filter (see Gabbiani et al., 1999), with the integral of the smoothed waveform scaled to equal the number of spikes in the trial. 

Data were plotted using Matlab and statistical tests were performed using STATISTICA (StatSoft, Tulsa, Oklahoma, USA). When analyzing spike number and peak firing rate samples we applied square root transformation over data and performed a Repeated measures ANOVA, which included planned comparisons. Gradient and intercept analysis of habituation regression curves was performed by a Factorial ANOVA.  Both ANOVAs were followed by Tukey's Honestly Significantly Different (HSD) post-hoc test. P-values of  0.05 were deemed statistically significant. Results are described as means ± standard deviations while standard errors (S.E.) were used for plotting.

SI references

I. Hen, A. Sakov, N. Kafkafi, I. Golani, Y. Benjamini, The dynamics of spatial behavior: how can robust smoothing techniques help?, J. Neurosci. Methods 133: 161 (2004).

C. W. Gardiner, Handbook of Stochastic Methods: for Physics, Chemistry and the Natural Sciences, 3rd ed., Springer, Berlin (2004).


Table S1

Tested radii (cm)	Front visual field	Back visual field	
	D	p-value	D	p-value	
1//2	1.00	1.79E-133	1.00	1.79E-133	
2//3	0.99	6.00E-130	0.98	3.33E-128	
3//4	0.37	2.59E-18	0.58	1.07E-45	
4//5	0.28	4.17E-11	0.35	1.01E-16	
5//6	0.28	1.31E-10	0.29	2.33E-11	
6//7	0.20	9.53E-06	0.28	1.31E-10	
7//8	0.16	5.64E-04	0.23	1.14E-07	
8//9	0.13	0.0113	0.13	0.0086	
9//10	0.09	0.1671	0.11	0.0613	
10//11	0.05	0.7762	0.06	0.6397	
11//12	0.07	0.3823	0.06	0.5706	
12//13	0.05	0.7762	0.07	0.5039	
Saturation of the visual signal in the front and the back visual field. A set of two-samples Kolmogorov-Smirnov tests between sequential radii revealed that the curves in Figure S4 significantly differ from one another between radii of 1-9 cm. Beyond the radius of 9 cm no significant difference was found.


Table S2
(A)

State	Frequency	
1 – low movement	7%	
2 – ordered CCW	32%	
3 – unordered	10%	
4 – ordered CW	32%	
5 – transitional (not assigned)	19%	

(B)
State	To 1	To 2	To 3	To 4	
From 1		40%	20%	40%	
From 2	95%		5%		
From 3	80%	10%		10%	
From 4	95%		5%		

Approximating the simplified model as a four-state CTMC. (A) The relative time the system spends in each of the four states. Transitional frames are intermittent transitions while between states. (B) Matrix showing the probability of transition from state to state.


Supplementary material figures

Figure S1
The experimental setup: Several dozen locusts are placed in a plastic circular arena with a diameter of approximately 50cm.


Figure S2

Video analysis algorithm. (A) A hand-labeled frame in which animal were painted red and some of the background was painted blue. (B) the RGB content of labeled pixels. The two clusters are separated using a support vector machine. (C) All pixels in each frame are classified as either animal (red) or background (blue). (D) Labeled regions that fit certain size properties. (E) Numbered objects. (F) Voronoi cells associated with individual animals.


Figure S3

Experimental results: The distribution of walking speeds.


Figure S4
Experimental results. The optical flow in the front (A) and in the back (B) halves of the walking initiating animal's visual field. Each curve shows the average number of walkers at time t from a walking initiation event within a given distance from the animal that is starting to walk. For example, to generate the purple curve we listed all walking initiation events. Suppose that during event 1, animal k start walking at time . We counted the number of animals walking within a distance of 5 cm from animal k at time  and then averaged over all walking initiation events. Other distances we calculated in a similar manner. While in the front, a walking initiation is preceded by a reduction in the number of moving nymphs, an increase in the same parameter is seen in the back. In both halves of the visual field the signal saturates at around 9 cm, suggesting that above this radius no further visual information regarding the animal's surrounding is obtained.


Figure S5

Experimental results: Angles. (A) A histogram showing the distribution of angles in which animals that started walking due to a tactile stimulus were touched. (B) The angle between the head direction 10 frames before walking and the velocity vector five frames after walking started.


Figure S6

Experimental results. The time-evolution of the order parameter (blue) and fraction of walking animals (red) in the three experiments described in Table 1.The two variables are correlated with an average correlation coefficient of 0.38.

Figure S7

Detailed model results: The distribution of the average order parameter (A), average fraction of moving particles (B), and the correlation between the two (C) in 1500 simulations with length corresponding to 30 minutes of experiment time.


Figure S8

The distribution of waiting times between transitions in the 4-states CTMC approximation. (A) The detailed model. (B) The simplified model .x: state 1, o: states 2 and 4, +: state 3. Solid, dashed and dotted curves are a maximal likelihood fit to an exponentially distributed random variable from states 1, 2+4 and 3, respectively.


Figure S9

Detailed model results: Parameters for the diffusion equation describing the effective dynamics of the order parameter, showing three meta-stable states. (A) The drift, with three stable roots. (B) The diffusion coefficient,  has a maximum around zero. (C) The potential, , and (D) The probability density function,.


Figure S10

Variation of parameters in the detailed model. Top row: number of particles 10 (blue), 15 (red), 34 (green-experimental value), 100 (black). Middle row: Probability to start moving following touch  0.1 (blue), 0.2 (red), 0.3674 (green-experimental value), 0.4 (black), 0.5 (purple). Bottom row: Probability to start moving following the visual stimulus  0.02 (blue), 0.03 (red), 0.039(green-experimental value), 0.05 (black), 0.06 (purple).

Figure S11

Variation of parameters in the detailed model. Top row: Probability to stop moving following touch  0.3 (blue), 0.4 (red), 0.5083 (green-experimental value), 0.6 (black), 0.7 (purple). Middle row: . 10 (blue), 15 (red), 34 (green-experimental value), 50 (black),70 (purple). Bottom row: Interaction radius  3 (blue), 4 (red), 5 (green-experimental value), 6 (black), 7 (purple). As changes the average number of interacting neighbors, this is also equivalent to changing the interaction thresholds  and  .


Figure S12


Variation of parameters in the simplified model. Left column: effective drift, middle column: effective diffusion and right columns: effective potential. Top row: dependence on , Middle row: dependence on  and bottom row: dependence on .


Figure S13

Phase related differences in DCMD response. (A) The habituation observed in the elicited number of spikes was more substantial in solitarious nymphs than in gregarious ones (blue and red respectively). (B) Average number of spikes elicited by single and multiple stimuli for each phase. Gregarious (red): single object 28.64±4.31, multiple objects 48.32±7.61. Solitarious (blue): single object 27.87±5.28, multiple objects 30.31±5.19. *** Planned comparisons revealed a significant difference between the phases in the number of spikes elicited in response to movement of multiple objects and a difference in the response to single versus multiple approaches within the gregarious group (p<0.001).

Figure S14

A triple interaction between phase, stimulus type and habituation was not found. Habituation is presented separately for gregarious (dark line) and solitarious (bright line) animals and for each of the different visual stimuli, using regression lines fitted for the log-transformed number of spikes. Under all four visual conditions, the solitarious nymphs' regression lines were sharper in negative gradient.    


Movie captions

Movie S1. A short clip showing a 2 minutes example of an experiment in real time.
Movie S2. Stop and go motion in the field. Southern Israel, 2013.
Movie S3. Stop and go motion in the field. Southern Israel, 2013.
Movie S4. A short clip of an experiment with tracking results showing the build-up of motion. x5 speed-up.
Movie S5. A short clip of an experiment with tracking results showing synchronization as a preference to the clock-wise direction. x5 speed-up.
Movie S6. A short clip showing the switching between synchronized states in the detailed model.
